# Pyrrolysine-Inspired in Cellulo Synthesis of an Unnatural Amino Acid for Facile Macrocyclization of Proteins

**DOI:** 10.1021/jacs.3c01291

**Published:** 2023-04-26

**Authors:** Jingxuan Tai, Lin Wang, Wai Shan Chan, Jiahui Cheng, Yuk Hei Chan, Marianne M. Lee, Michael K. Chan

**Affiliations:** School of Life Sciences and Center of Novel Biomaterials, The Chinese University of Hong Kong, Hong Kong SAR 999077, China

## Abstract

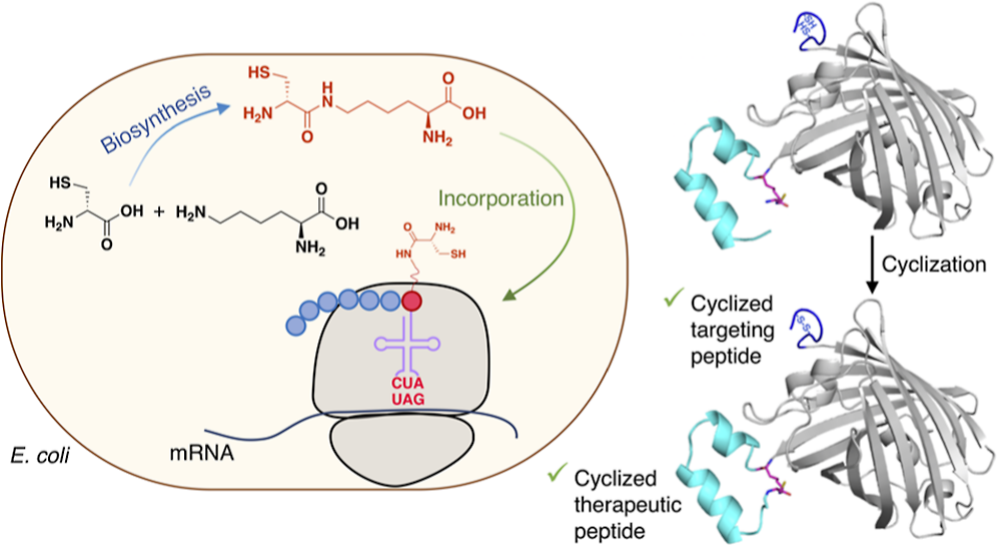

Macrocyclization has been touted as an effective strategy to enhance the in vivo stability and efficacy of protein therapeutics. Herein, we describe a scalable and robust system based on the endogenous biosynthesis of a noncanonical amino acid coupled to the pyrrolysine translational machinery for the generation of lasso-grafted proteins. The *in cellulo* biosynthesis of the noncanonical amino acid d-Cys-ε-Lys was achieved by hijacking the pyrrolysine biosynthesis pathway, and then, its genetical incorporation into proteins was performed using an optimized PylRS/tRNA^Pyl^ pair and cell line. This system was then applied to the structurally inspired cyclization of a 23-mer therapeutic P16 peptide engrafted on a fusion protein, resulting in near-complete cyclization of the target cyclic subunit in under 3 h. The resulting cyclic P16 peptide fusion protein possessed much higher CDK4 binding affinity than its linear counterpart. Furthermore, a bifunctional bicyclic protein harboring a cyclic cancer cell targeting RGD motif on the one end and the cyclic P16 peptide on the other is produced and shown to be a potent cell cycle arrestor with improved serum stability.

## Introduction

Protein and peptide therapeutics are important alternatives to small molecule drugs for the treatment of diseases due to their generally higher specificity and lower toxicity.^1^ One major limitation to protein/peptide-based therapeutics, however, is their rapid degradation by proteolytic enzymes in vivo leading to short serum half-lives, which adversely affect their efficacies.^1^ Cyclization has been shown to be a powerful approach to overcome this serum stability challenge,^2−5^ and cyclized proteins typically exhibit dramatically extended in vivo lifetimes, as well as improved binding affinities due to reduction in the entropic cost of binding.^6,7^

Different approaches have been developed to cyclize polypeptides, including the placement of cysteine residues in close spatial proximity to promote disulfide bond formation, the use of chemical cross-linking agents,^8^ the use of split inteins to produce head-to-tail cyclic peptides,^9^ and the site-specific incorporation of noncanonical amino acids (ncAAs) with distinct functionalities for cyclization mediated by an orthogonal tRNA/aminoacyl-tRNA synthetase pair.^10−12^ One key advantage of the latter approach is that its site-specific nature enables bioorthogonal functionalities to be placed at virtually any position within a protein and thus allows for the engraftment of multiple cyclic subunits on the same protein, and the generation of bi- or tri-cyclic proteins against distinct targets.^13^

**Figure 1:**
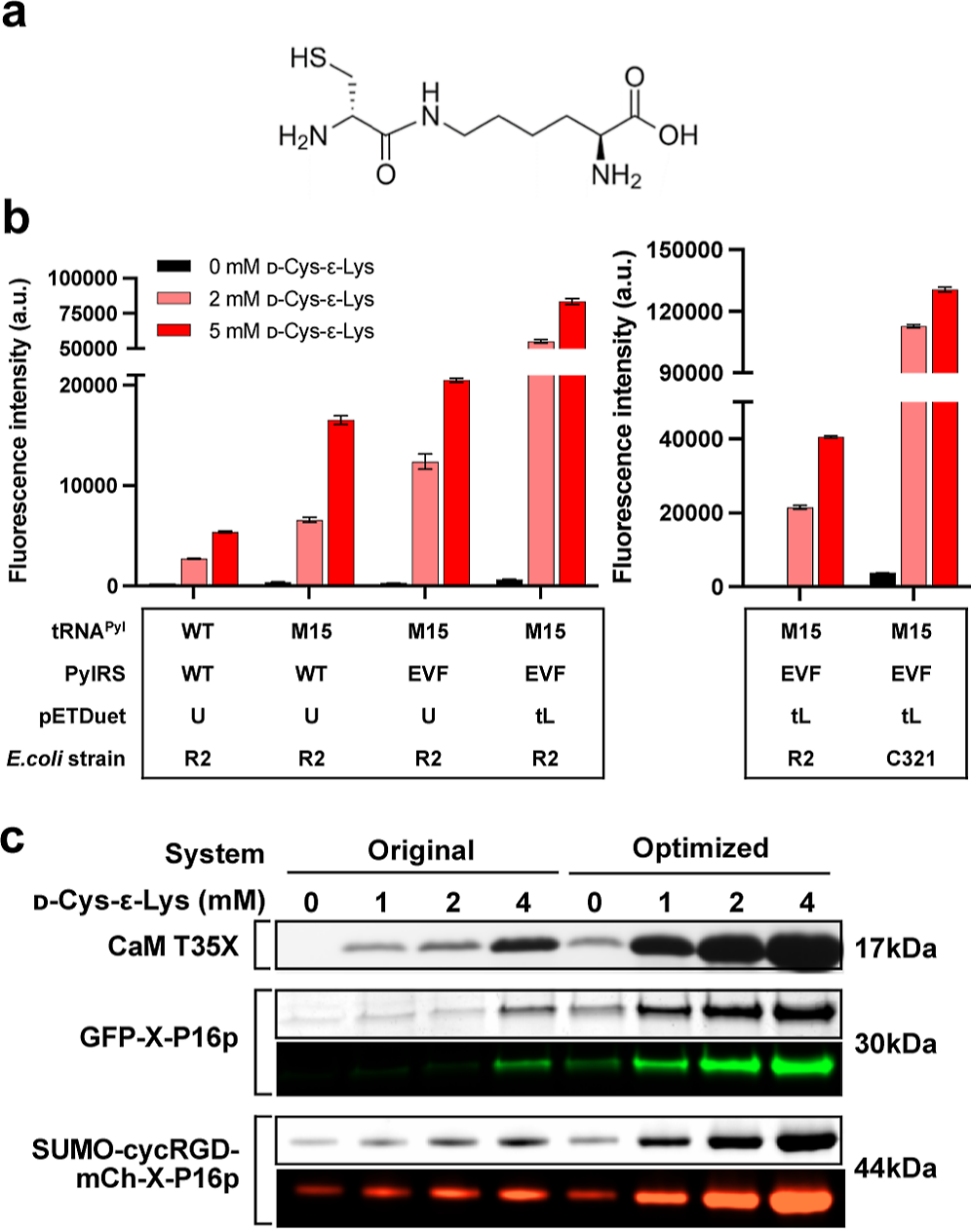
Optimization of the d-Cys-ε-Lys readthrough system. (a) Chemical structure of d-Cys-ε-Lys. (b) mCherry readthrough assay comparing the original and the optimized UAG readthrough system. The lysine codon at position 55 in the mCherry gene was mutated to TAG, and the medium was supplemented with 0, 2, or 5 mM d-Cys-ε-Lys. Data represent mean fluorescence intensity ± standard error of the mean (*n* = 3). WT: wild type. M15: tRNA^M15^. U: unmodified pPylST. tL: modified pPylST with the two T7*lac* promoters replaced by P_tac_ and PL_lacO1_, respectively. R2: Rosetta 2 (DE3). C321: C321.ΔA.M9adapted. (c) Comparison of the original UAG readthrough system and the optimized one for d-Cys-ε-Lys incorporation into different recombinant proteins. X represents d-Cys-ε-Lys. CaM is short for calmodulin.

Our laboratory has previously reported the use of a cysteine-containing pyrrolysine analogue, d-cysteinyl-*N*^ε^-l-lysine (d-Cys-ε-Lys, abbreviation: X, Figure 1a), genetically encoded by the amber UAG codon, to cyclize a RGD motif appended to an mCherry protein via intein-mediated native chemical ligation (NCL).^10,14^ The incorporation of d-Cys-ε-Lys into the recombinant protein was enabled by the introduction of *Methanosarcina mazei* pyrrolysyl-tRNA synthetase/tRNA^Pyl^ (PylRS/tRNA^Pyl^) pair into *Escherichia coli* (*E. coli*) cells.^15^ This study demonstrated the versatility of the pyrrolysine technology for the generation of a tadpole-like protein bearing a branched cyclic peptide. Significantly, given that the resultant cyclic structure was held together by an isopeptide bond, it was stable in the reducing environment of the cytosol leading to enhanced stability against proteolysis as demonstrated in vitro.^10^

Nevertheless, there were several lessons to be learnt from this proof-of-concept study. While we could obtain cyclized proteins with high purity,^10^ the product yields were low. Several factors were observed to impact production yield, the most notable being an inefficient d-Cys-ε-Lys incorporation, an oft-mentioned challenge in most ncAA-utilizing systems, as well as incomplete and inefficient cyclization of the protein. Furthermore, in the process of producing an adequate amount of the cyclized protein for investigation, we realized that the cost of the chemically synthesized pyrrolysine analogue could quickly pile up, making the system economically unviable. Thus, we sought to overcome these technological hurdles as an important step toward the practical development of new cyclic peptide drugs based on the pyrrolysine technology.

In this report, we describe the development of a simple and efficient method for the in vivo biosynthesis of the pyrrolysine analogue d-Cys-ε-Lys in *E. coli* cells that can easily be extended to other analogues. The resultant in cellulo-synthesized d-Cys-ε-Lys was concomitantly incorporated into ribosomally synthesized polypeptides for the production of d-Cys-ε-Lys-containing proteins mediated by an optimized pyrrolysine translational system. To demonstrate the effectiveness of this approach, a 23-mer peptide derived from the tumor suppressor protein P16 (P16p) was chosen as a model system for cyclization.

P16 is an endogenous inhibitor of the cyclin-dependent kinase 4/6 (CDK4/6). By blocking the formation of the cyclin D-CDK4/6 complex responsible for the phosphorylation of pRb, the transition of cell cycle from G1 phase to S phase is halted.^16^ Three small molecule inhibitors of CDK4/6 have been approved by the FDA for the treatment of multiple cancer types, though toxicity remains a concern.^17^ The selected P16p, which encompasses the P16-interacting motif with CDK4/6,^18,19^ has been shown to similarly inhibit pRb phosphorylation and in turn G0/G1 cell cycle progression, resulting in the suppression of cancer cell proliferation in both in vitro and in vivo studies.^18,20,21^

Given the previously observed incomplete and slow rate of cyclization of the C-terminal RGD-containing peptide,^10^ a major motivation in choosing the P16p peptide—in addition to its clinical significance—was our conjecture that the conformation flexibility of the polypeptide might have hindered the d-Cys-ε-Lys and the C-terminal thioester in achieving the optimal configuration for NCL. Thus, we hypothesized that a peptide fragment known to adopt a structural motif, such as the P16p that forms a helix-turn-helix structure (Figure S1) might help to facilitate cyclization. In their seminal studies on protein cyclization via NCL, Muir et al.^22^ had demonstrated that the rate of cyclization of the WW domain of the human Yes kinase-associated protein was greatly enhanced when its N- and C-termini were placed in close proximity. We thus hypothesized that cyclization of a pre-formed motif would be more efficient and that its successful cyclization would confer the P16p peptide with superior stability and potency, thereby enhancing its therapeutic potential in the treatment of cancer.

## Results

### Optimization of the d-Cys-ε-Lys Readthrough System

Our initial effort to optimize the UAG readthrough system focused on enhancing the d-Cys-ε-Lys incorporation into proteins. Since d-Cys-ε-Lys is not the natural substrate for wild-type pyrrolysyl-tRNA synthetase (PylRS), we rationalized that improving the substrate specificity or activity of PylRS for d-Cys-ε-Lys might improve its catalytic efficiency, thereby leading to more efficient d-Cys-ε-Lys incorporation. To this end, directed evolution was employed to evolve a PylRS mutant that could specifically recognize d-Cys-ε-Lys and display improved efficiency in its genetic incorporation.

To generate the PylRS mutant library, a plasmid harboring the genes encoding the wild-type PylRS, tRNA^M15^—a more stable variant of tRNA^pyl^ with demonstrated improved amber suppression efficiency,^23,24^ kanamycin resistance protein aphA-3, and mCherry protein was constructed. The latter two proteins were each engineered with a permissive UAG codon to facilitate a two-tier selection and screening protocol for the rapid detection of active PylRS mutants and their corresponding d-Cys-ε-Lys incorporation activity. Here, the mutants were first subjected to positive selection based on the library-transformed bacterial cells to survive under kanamycin selection pressure contingent on d-Cys-ε-Lys incorporation into the kanamycin resistance polypeptide. The survival clones were then screened for their strong mCherry fluorescence enabled by the readthrough of the UAG codon in the mCherry transcripts. After iterative mutagenesis and screening, a triple mutant PylRS carrying G14E, C348V, and S451F mutations (PylRS^EVF^) that exhibited improved readthrough efficiency was identified (Figure 1b, left panel).

Computational modeling was then performed to ascertain how these three mutations might impact substrate binding. Of the three mutated residues, the residue C348 has been reported to be critical for the binding of substrates.^25^ Previous studies on the structure of *Mm*PylRS revealed that C348 together with Y306, Y384, V401, and W417 forms a deep hydrophobic pocket (Figure S3a, upper left panel) and is among the key residues involved in the recognition and binding of pyrrolysine and its analogues.^26,27^ Presumably, the substitution of the polar cysteine with the more hydrophobic valine enhances the hydrophobicity of the binding pocket as verified by CHIMERA,^28^ thereby improving its affinity for d-Cys-ε-Lys (Figure S3a). The other two mutated residues, G14 and S451, are located in the N-terminal tRNA binding domain and C-terminal tRNA minimal core binding surface of PylRS, respectively (Figure S3b). Subsequent optimization of other parameters, including the replacement of T7 with T7lac promoters resulted in a further yield improvement, with a net ∼20-fold increase in mCherry fluorescence compared to protein expression and readthrough using WT PylRS and tRNA^Pyl^.

To further improve the yield of d-Cys-ε-Lys containing-protein produced, we next explored the use of the genetically recoded *E. coli* strain (C321.ΔA.M9adapted)^29^ whose gene-encoding release factor 1 was deleted. Using this strain brought a further 5.3-fold improvement based on the mCherry fluorescence (Figure 1b). The robustness of this final optimized d-Cys-ε-Lys readthrough system was subsequently tested. Notably, consistent enhancement in d-Cys-ε-Lys incorporation and readthrough efficiency of proteins of different sizes was achieved, based on the much higher level of full-length proteins produced (Figure 1c).

### In Vivo Biosynthesis of Pyrrolysine Analogue d-Cys-ε-Lys

Most current studies on the use of ncAAs to produce ncAA-containing proteins rely on the exogenous supplementation of ncAAs chemically synthesized with the functionalities of interest. Such an approach, however, is expensive and not sustainable for large scale production of ncAAs-containing proteins. A more economical and practical strategy would be to produce the ncAAs of interest from basic carbon sources or amino acids (which can be purchased cheaply) in a workhorse such as *E. coli*, which are then directly incorporated into proteins in response to the amber codon.

**Figure 2:**
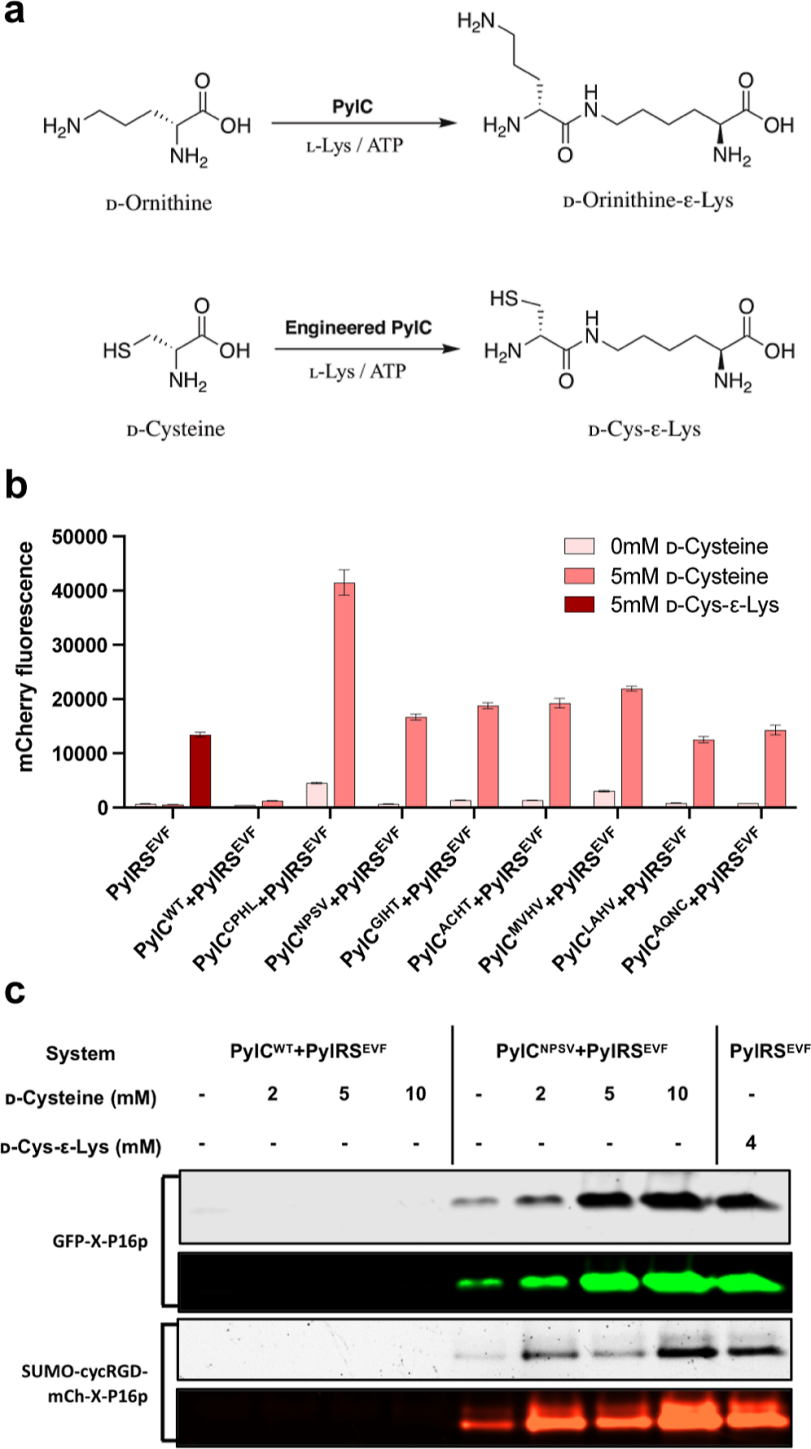
Engineering PylC for in vivo synthesis of d-Cys-ε-Lys. (a) Schematic representation of the enzymatic function of engineered PylC. (b) Screening of putative PylC mutants that could recognize d-cysteine and catalyze the production of d-Cys-ε-Lys based on mCherry fluorescence. Saturation mutagenesis was performed on four residues: S177, E179, D233, and T256 of PylC. Data represent mean fluorescence intensity ± standard error of the mean (*n* = 3). (c) Comparison of the wild type PylC (PylC^WT^) and the evolved PylC mutant (PylC^NPSV^) in the production of d-Cys-ε-Lys for its incorporation into UAG-containing proteins at different concentrations of d-cysteine. Sample readthrough was benchmarked against the readthrough protein produced by exogenous supplementation of 4 mM d-Cys-ε-Lys.

In this regard, the elucidated pyrrolysine biosynthesis pathway^30^ offers us a blueprint for the establishment of an endogenous biosynthesis machinery of ncAAs, in our case d-Cys-ε-Lys. Pyrrolysine (Pyl) is generated by the action of PylB, PylC, and PylD enzymes. Here, lysine is first converted by PylB to (2*R*,3*R*)-3-methyl-ornithine, which is then coupled to a second lysine by PylC to form l-lysine-*N*^ε^-3*R*-methyl-d-ornithine (Lys-*N*^ε^-3MO), a precursor of pyrrolysine. PylD then converts the terminal ornithyl amine to a carbonyl, which then reacts with an amine to form the pyrroline group. Previous studies have shown that Pyl could be synthesized in the absence of PylB.^30^ These findings prompted us to examine the Pyl biosynthesis pathway and wonder whether PylC, which catalyzes the d-ornithinyl-l-lysine ligation reaction, could be re-engineered to couple other amino acids, such as d-cysteine (d-Cys), to l-lysine to form d-Cys-ε-Lys (Figure 2a). The complex structure of *Methanosarcina barkeri* PylC with d-ornithine has been determined^31^ and showed that four active site residues (S177, E179, D233, and T256) were important for d-ornithine binding. Thus, to evolve a PylC mutant that could take d-Cys as the substrate, we performed saturation mutagenesis on these four residues to create a mutant library, which was then used to coexpress with the optimized PylRS^EVF^/tRNA^M15^ in culture medium supplemented with d-Cys for downstream screening assay as described above. We subsequently identified a mutant PylC^NPSV^ (S177N, E179P, D233S, and T256V) that survived kanamycin selection and showed high mCherry fluorescence with the least fluorescent background (Figure 2b).

To understand the basis of this switch in specificity, computational models of *Mm*PylC^WT^ and *Mm*PylC^NPSV^ bound to d-Cys-ε-Lys were generated using the program CNS.^32,33^ Based on these models, it appears that mutations of E179P and T256V convert the original hydrophilic d-ornithine binding site to a hydrophobic pocket (Figure S4a) that makes binding of the charged amino group of the d-ornithine side chain less favorable. On the other hand, mutations of the residues T256 and S177 to T256V and S177N in PylC^NPSV^ result in van der Waals interactions with the thiol group of d-Cys-ε-Lys based on analysis using CHIMERA (Figure S4b). It is likely that this combination of weaker binding of d-ornithine-ε-Lys and improved binding of the d-Cys-ε-Lys leads to the observed switch in substrate specificity.

To evaluate the readthrough efficiency using *in cellulo* biosynthesized d-Cys-ε-Lys as opposed to exogenous addition of its chemically synthesized counterpart, different protein constructs were tested and the results indicated that at 5 mM or higher d-Cys, PylC^NPSV^ in cooperation with PylRS^EVF^/tRNA^M15^ could achieve a readthrough efficiency comparable to that of the exogenous addition of 4 mM chemically synthesized d-Cys-ε-Lys (Figure 2c). The successful *in cellulo* biosynthesis of d-Cys-ε-Lys was subsequently confirmed via the identification of the d-Cys-ε-Lys-containing peptide fragment by LC–MS/MS analysis of the corresponding trypsin-digested protein (Figure S5).

### Structure-Inspired Cyclization of P16p

Having resolved the issue of readthrough efficiency that in part contributes to the poor cyclized protein yield, we then moved to work on improving the cyclization step. In our previous study, we observed slow and incomplete cyclization of an RGD motif placed on the C-terminus of an mCherry fusion protein.^10^ At that time, we speculated that the low yield was due to the multiple degrees of freedom of the reacting groups. Further supporting this observation was the insights from other studies that the relative spatial positions of the N- and C-termini of the peptide to be cyclized greatly affect cyclization rate and yields.^34−36^ Thus, we hypothesized that utilizing structured fragments where the d-Cys-ε-Lys and C-terminal thioester are better spatially localized for cyclization might lead to enhanced cyclization efficiencies. Many natural cyclic proteins possess such pre-formed motifs within their sequence, and we therefore sought to identify a therapeutic protein from which we could extract the relevant structural element and incorporate it into our designed construct for cyclization. Toward this end, we were drawn to the tumor suppressor P16 protein whose binding sequence to the CDK4/6 contains a helix-turn-helix structure (Figure S1).^19^ P16 exerts its tumor-suppressor effect via its action as a CDK4/6 inhibitor that prevents the phosphorylation of retinablastoma (Rb) resulting in G0/G1 cell cycle arrest. The P16 binding sequence to CDK4/6 has been characterized and is found to be a 20-mer peptide encompassing residues 84–103 (P16p).^18^ Thus, we surmised that cyclization of the P16p should enhance its binding affinity and cellular stability, thereby its therapeutic potential.

**Figure 3:**
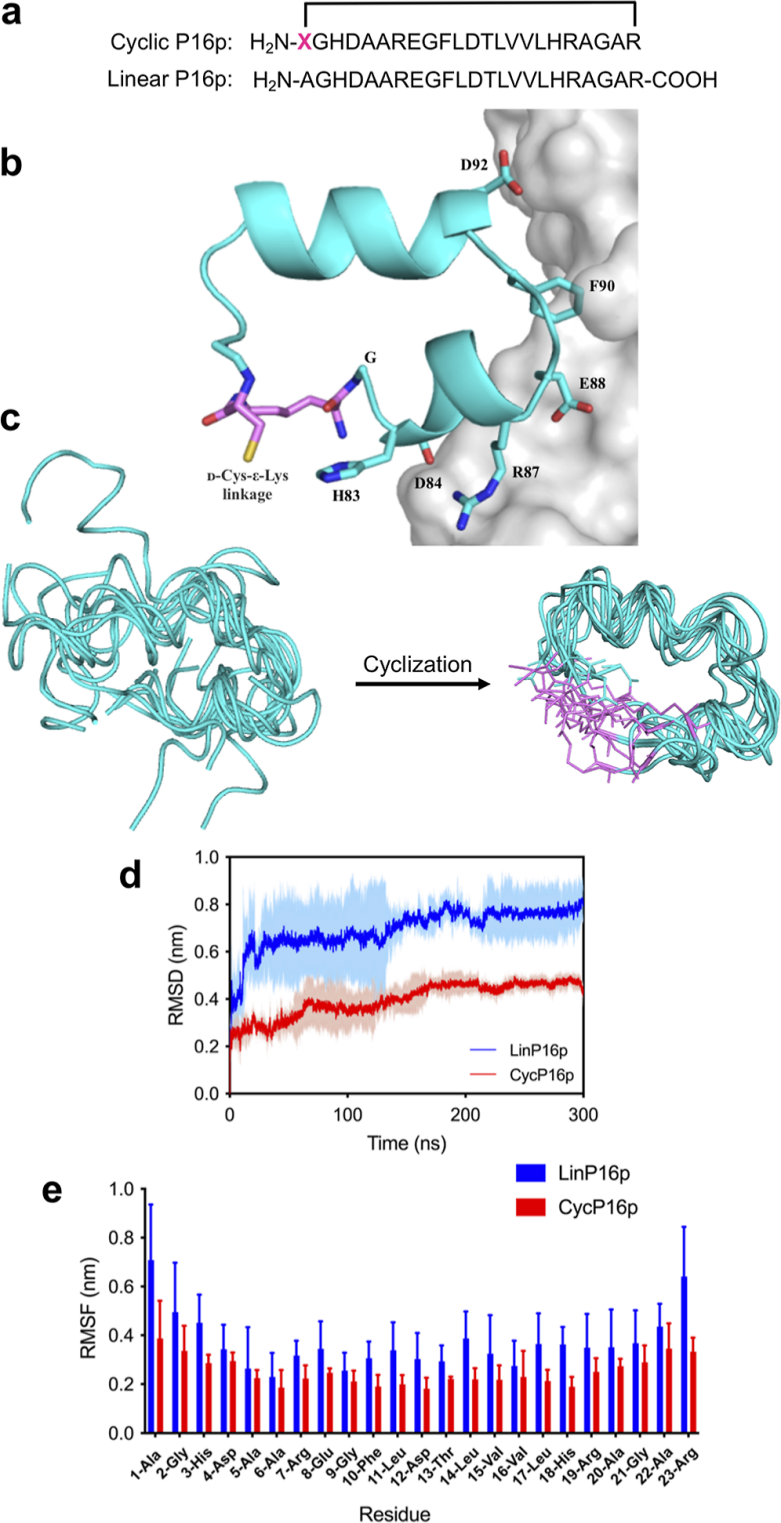
Computational analysis of cyclized P16 peptide. (a) Amino acid sequences of cyclic P16 peptide (cycP16p) showing site of d-Cys-ε-Lys incorporation (denoted by X) and linear P16p. The black bracket denotes d-Cys-ε-Lys linkage-mediated cyclization. (b) Model of cycP16p interacting with CDK6. The structure of p16p (cyan) and CDK6 (gray) complex was derived from PDB: 1BI7. CDK6-interacting residues were labeled with their numbers in P16 protein. (c) Comparison of MD simulation results between linear P16p (LinP16p) (left) and cycP16p (right). Figures are superposition of 10 rounds of LinP16p (left) and CycP16p (right) MD simulations at 10 ns. (d) RMSD and (e) RMSF profiles of LinP16p and CycP16p in 300 ns MD simulation. Results were calculated based on backbone atoms. Error bars present standard deviation (SD) of 3 replicate simulation runs and were plotted as shaded area in the RMSD profile.

Since the P16/CDK6 complex structure is available, we used it to help guide us in designing the d-Cys-ε-Lys incorporation site and performed molecular simulation to verify our design strategy. Based on structural analysis, the site of incorporation was designed to be located at the N-terminus of P16p, which was extended by one more residue to include the 83rd residue histidine. In addition, an N-terminal glycine was added to enable d-Cys-ε-Lys to be spatially close to the C-terminal thioester generated during cyclization (Figure 3a,b). To obtain visual insights of the designed cyclic P16 peptide, the corresponding molecular model was built using Pymol, which showed that the d-Cys-ε-Lys-linkage occurred on the opposite side of the P16-CDK4/6 interface (Figure 3b). Molecular dynamics simulations were then performed on this peptide as well as its linear counterpart to evaluate their structural stability. In simulation, linear P16p (LinP16p) displayed significant higher structural flexibility, in particular at the ends of the two helices, while the cyclic P16p exhibited a much more stable conformation with minimal changes from the reference structure (Figure 3c, Supporting Information Videos S1 and S2). This observation was corroborated by their respective RMSD and RMSF profiles, which depicted much larger amplitude fluctuations for the linear P16p compared with those of the cyclic P16p (Figure 3d,e). These results appeared to support the notion that cyclization would benefit P16p, at least in terms of enhancing its stability given the reduction in configurational entropy. We therefore proceeded to validate these results experimentally.

**Figure 4:**
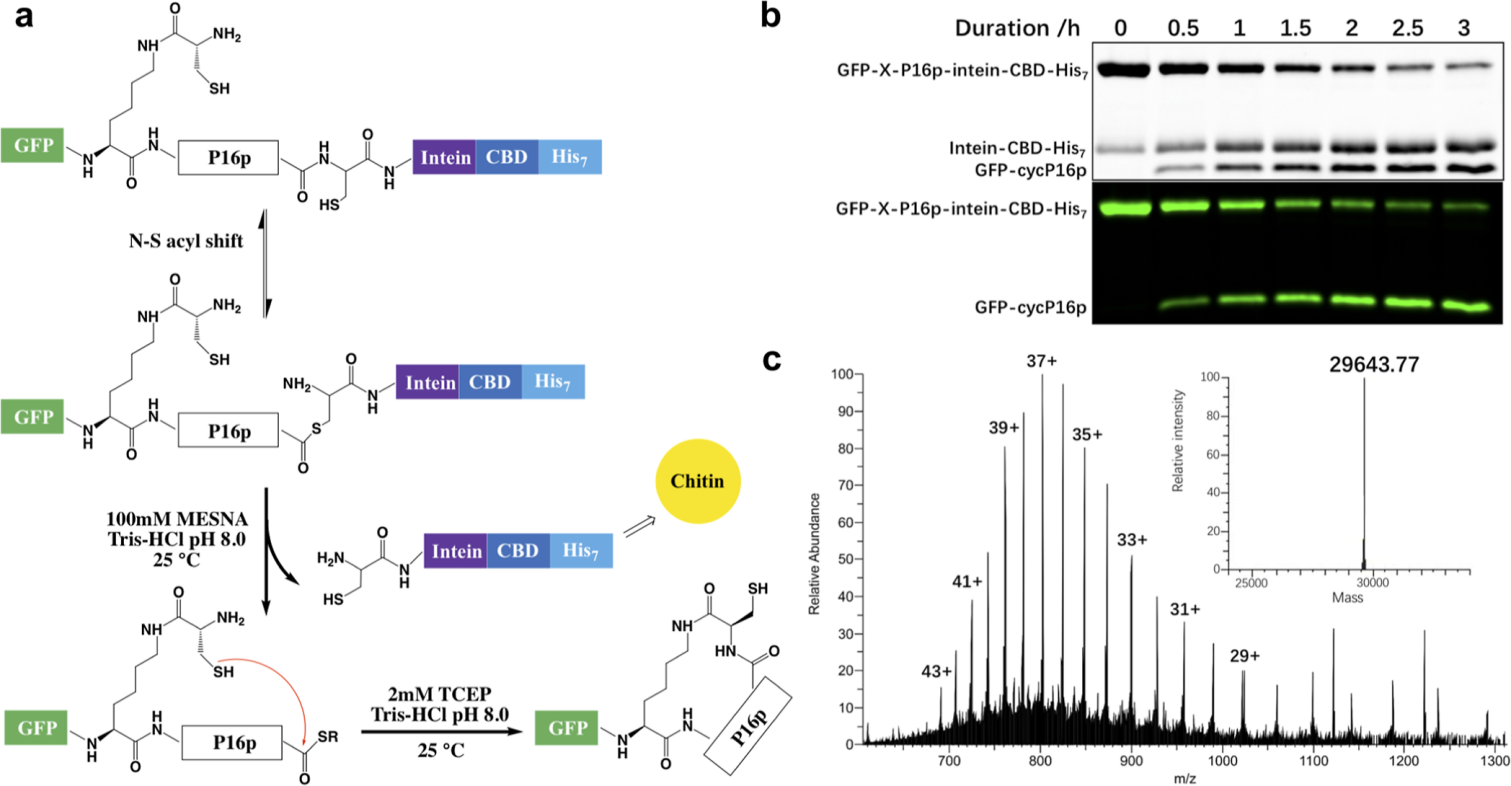
Intramolecular cyclization of GFP-P16p. (a) Schematic illustration of the protein construct GFP-X-P16p-intein-CBD-His_7_ and mechanism of d-Cys-ε-Lys-based protein cyclization. (b) SDS–PAGE of reaction samples taken at different time points during the cyclization of GFP-X-P16p. Shown are gels stained by coomassie blue (upper) and detected by in-gel GFP fluorescence (lower). (c) Deconvoluted mass spectrum of GFP-cycP16p obtained by ESI-Orbitrap mass spectrometry.

The aforementioned P16p design was incorporated into a protein construct comprising a green fluorescent protein GFP, the P16p, an intein for thioester generation, and a CBD-His_7_ tag for affinity purification (GFP-X-P16p-intein-CBD-His_7_) (Figure 4a). Utilizing the optimized PylRS^EVF^/tRNA^M15^ pair and the engineered PylC^NPSV^, ∼19 mg of purified d-Cys-ε-Lys-containing GFP-X-P16p-intein-CBD-His_7_ protein could be obtained from a 200 mL cell culture grown in medium supplemented with 5 mM d-Cys. This product yield represented a 10-fold improvement over the unoptimized system. Then, the intein-mediated cleavage of GFP-X-P16p-intein-CBD-His_7_ was initiated by the addition of MESNA and, encouragingly, was completed within 3 h (Figure 4b), as indicated by the GFP signal of both full length and cleaved fragment leveled off after the 2.5 h time point. The resultant cyclization product was subsequently confirmed by mass spectrometric analysis (MS) to be the cyclized form of GFP-P16p (GFP-cycP16p) with a monoisotopic mass of 29,643.77 (theoretical mass is 29,642.72) (Figure 4c). Moreover, the MS analysis indicated that the GFP-cycP16p was the dominant component in the cyclization reaction mixture. Compared to the pyrrolysine-inspired cyclization of RGD in our previous study,^10^ which took >96 h and reached only 58% completion, the facile cyclization of P16p in under 3 h was nearly quantitative—a drastic enhancement in efficiency and completion. These results thus validated our hypothesis that preformed structural motifs can be used to promote facile cyclizations.

### In Vitro Binding Studies of Cyclized P16p to CDK4

**Figure 5:**
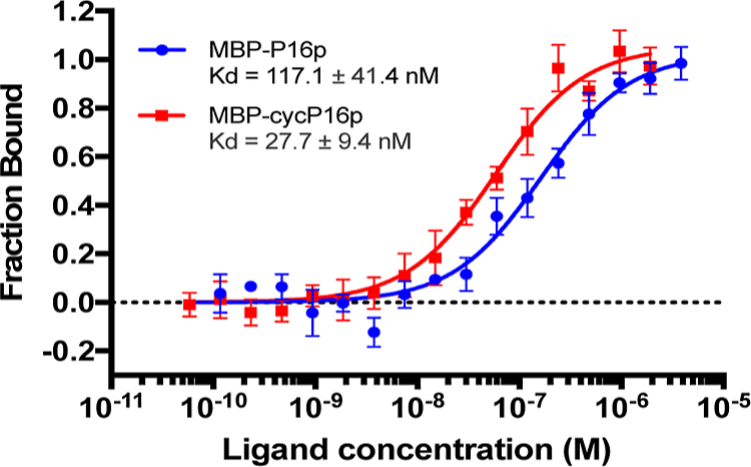
Cyclic P16p exhibits higher CDK4-binding affinity than its linear counterpart. Binding curves from MST assays of MBP-P16p and MBP-cycP16p with GST-CDK4 are shown. Error bars present standard deviation (SD) of three replicate measurements.

To evaluate the impact of cyclization on the binding affinity of P16p with CDK4, analytical size exclusion chromatography (SEC) and microscale thermophoresis (MST) were performed. SEC analysis showed that GFP-cycP16p interacted with GST-CDK4 to form a complex (Figure S8a). In the MST binding assay, the GFP tag of the P16p constructs was replaced with an MBP tag to avoid fluorescence interference. Pull-down assays were conducted to verify that the resultant constructs could still capture CDK4 from MCF-7 cell lysate (Figure S8b). Subsequent MST binding studies showed that the cyclized MBP-P16p protein (MBP-cycP16p) exhibited four-fold higher binding affinity with GST-CDK4 (*K*_d_ = 27.7 ± 9.4 nM, Figure 5) compared to its linear counterpart MBP-P16p (*K*_d_ = 117.1 ± 41.4 nM, Figure 5).

### Generation and In Vitro Characterization of Bifunctional Bicyclic cycRGD-mCh-cycP16p

Having shown that the pre-formed motif strategy was effective in enhancing cyclization efficiency and that the cyclized version of P16p exhibited much higher binding affinity than its linear counterpart, we asked ourselves whether we could further improve the cyclized protein’s therapeutic potential, such as equipping it with a cyclic RGD for cancer cell targeting. We envisioned a dumbbell-like protein with the cyclic RGD at one end, acting as a tumor-homing device that specifically binds to αvβ3 integrin receptors often overexpressed on cancer cells, and the cyclized therapeutic agent at the other end acting on intracellular target to elicit anti-cancer response.

**Figure 6:**
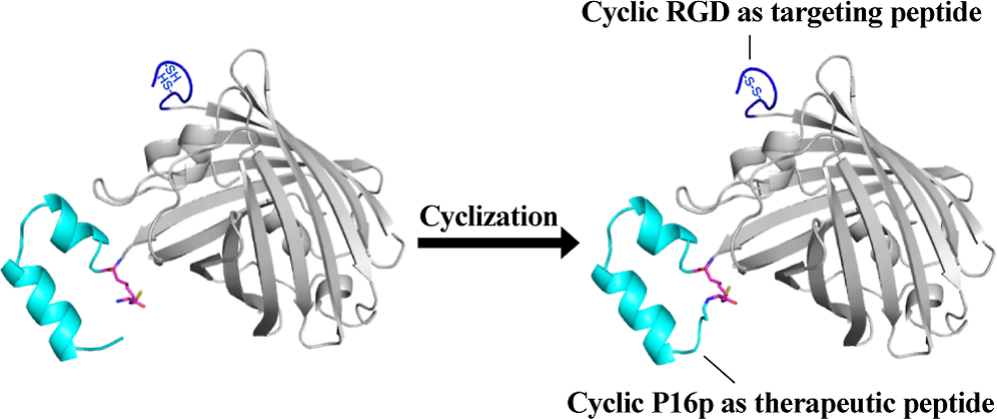
Model of the bicyclic cycRGD-mCh-cycP16p protein. RGD motif (marine blue) fused to the N-terminus of mCherry (gray) was cyclized by the formation of the disulfide bond between two cysteines. P16 peptide (cyan) fused to the C-terminus of mCherry was cyclized by the d-Cys-ε-Lys-based cyclization method.

To this end, a protein construct comprising an N-terminal disulfide-based cyclic RGD motif^37^ (cycRGD) to enhance cellular specificity and uptake via integrin binding, an mCherry protein to facilitate improved confocal imaging,^38^ the P16p described above and the intein-CBD-His_7_ tag (cycRGD-mCherry-X-P16p-intein-CBD-His_7_, Figure S6) was expressed, purified, and cyclized following similar procedures described for GFP-cycP16p and MBP-cycP16p. For the cyclization of the N-terminal RGD, the protein was air-oxidized for 24 h to produce bicyclic cycRGD-mCh-cycP16p (Figure 6). The cellular uptake and distribution of the cycRGD-mCherry bearing linear uncyclized p16p (cycRGD-mCh-P16p) and the cyclized P16p (cycRGD-mCh-cycP16p) were then evaluated by incubating the individual proteins with MCF-7 breast cancer cells for 20 h. Significant mCherry fluorescence was detected intracellularly in both treated cells (Figure S9) as indicated by sectional scanning using confocal laser scanning microscopy.

### Effect of Cyclization on the Inhibition and Stability of P16 Peptide

**Figure 7:**
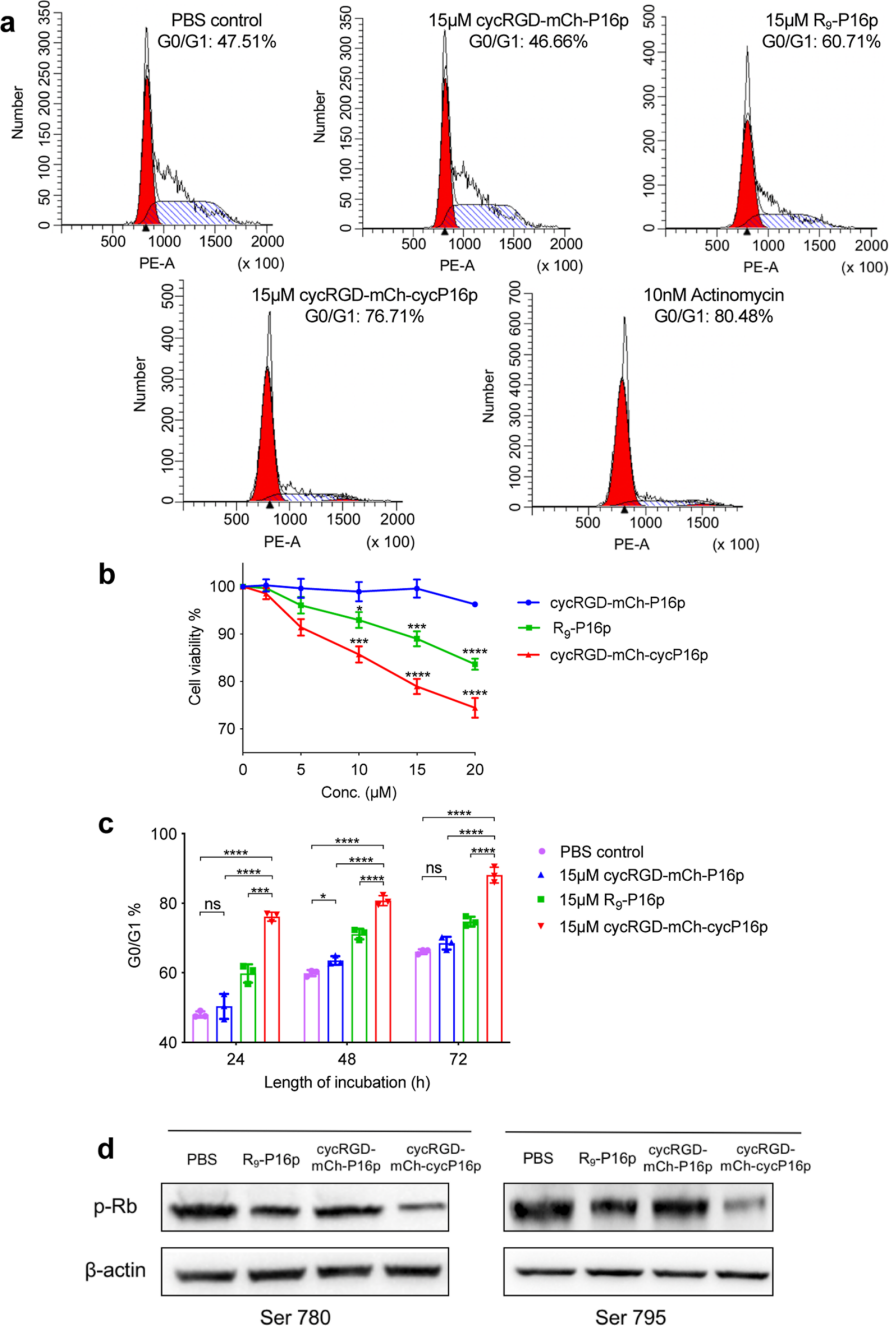
Effects of cycRGD-mCh-cycP16p on MCF-7 cells. (a) MCF-7 cells were exposed to different treatments for 24 h followed by cell cycle analysis on a BD flow cytometer. 10 nM actinomycin was included as the positive control. (b) MCF-7 cells after 24 h treatment with different peptides. Cell numbers were normalized to the PBS control group. Data are presented as the mean ± SD (*n* = 3). Statistical significances versus cycRGD-mCh-P16p group were shown. (c) Percent of arrested MCF-7 cells at the G0/G1 phase after exposure to different treatments at different time points. Error bars present standard deviation (SD) of three replicate measurements. *P* values are calculated by one-way ANOVA test. *p < 0.05, ***p < 0.001, ****p < 0.0001, ns, not significant. (d) Western-blot analysis of the phosphorylation status of Rb in MCF-7 cells exposed to different treatments using anti-pRb antibodies.

Having verified that both the linear and cyclized cycRGD-mCh-P16p could enter cells, we proceeded to investigate the ability of these proteins in halting cell cycle progression, and more importantly whether cyclization of the P16p could indeed endow cycRGD-mCh-cycP16p with enhanced cell cycle arrest ability, and in turn a more potent inhibitory effect on cancer cell growth. MCF-7 cells were treated with different concentrations of linear or cyclic cycRGD-mCherry-P16p. A linear P16p fused to the well-known poly-arginine cell penetrating sequence (R_9_-P16p) was also included for comparison. Flow cytometric analysis of the different groups of treated cells revealed that, at 15 μM concentration, cycRGD-mCh-cycP16p was the most potent cell cycle arrestor (76.71%), and compared favorably to R_9_-P16p, which was moderately effective (60.71%), while the linear counterpart cycRGD-mCh-P16p had a minimal effect on G0/G1 phase arrest (46.66%) (Figure 7a). Similar results were observed in the cell proliferation assay in which cycRGD-mCh-cycP16p exerted the strongest inhibition on MCF-7 cell growth compared with R_9_-P16p and cycRGD-mCh-P16p at the concentrations tested (Figure 7b). These enhanced performances of cycRGD-mCh-cycP16p could be attributed in part to its higher affinity binding to CDK4 in MCF-7 cells brought upon by the cycP16p subunit as indicated by the binding constant obtained from MST (Figure 5).

Given that cyclization has been used extensively to enhance the serum stability of peptides and proteins, the stability of cycRGD-mCh-P16p was evaluated by monitoring its ability to arrest cell cycle for an extended period of up to 72 h. This time course study indicated that the cycRGD-mCh-cycP16p retained its ability to induce G0/G1 cell cycle arrest even after prolonged exposure to serum-supplemented culture medium as shown by the approximately 88% of arrested cells at 72 h (Figures 7c and S10). By comparison, the linear cycRGD-mCh-P16p was not able to induce meaningful cell cycle arrest. These results were further verified by interrogating the phosphorylation status of specific sites on retinoblastoma (Rb) known to be associated with G1-to-S phase transition of the cell cycle in the differentially treated MCF cells. Western blot analysis using phosphor-specific anti-Rb antibodies for serine 780 and serine 795—the two residues whose phosphorylation will activate cell progression and inactive cell cycle arresting function of pRB, respectively—revealed significantly reduced levels of phosphorylation at the indicated sites in MCF-7 cells treated with cyclic cycRGD-mCh-cycP16p compared with those treated by the other two linear P16p’s (Figure 7d). Collectively, the results suggest that cyclization enhances the potency of P16p as a CDK4/6 inhibitor due in part to enhanced serum stability and binding affinity with CDK4. Notably, the use of d-Cys-ε-Lys allowed for the generation of an iso-peptide-linked cyclic P16p subunit that is more resistant to proteolysis and stable in the reducing cytosol—key features critical to the development of peptide therapeutics.

## Discussion

Recent advances have seen the expansion of the application of noncanonical amino acids (ncAAs) from basic science into the development of therapeutic entities. Despite its expanding role and importance, the low incorporation efficiency of the ncAA substrate and, consequently, low yields of the modified target protein remain a major challenge to ncAA-mediated applications. In this study, a multipronged approach was employed to improve the yield of d-Cys-ε-Lys-containing proteins. This includes optimization of the ncAA incorporation system via the replacement of tRNA^pyl^ with tRNA^M15^ to improve amber suppression, the engineering of a PylRS variant with tailored d-Cys-ε-Lys specificity to enhance ribosomal incorporation of d-Cys-ε-Lys, and the creation of an expression plasmid with promotors optimized for the production of ncAA-containing proteins in the genetically recoded C321 *E. coli* strain. Every optimization step resulted in an increase in the corresponding product yield and ultimately contributed to the overall improved efficiency (Figure 1b). At the same time, this optimized incorporation system laid the groundwork for the subsequent establishment of an in vivo system for the endogenous biosynthesis of the ncAA d-Cys-ε-Lys and the corresponding d-Cys-ε-Lys-containing proteins.

Another major hurdle is the production of the ncAA to be incorporated. To date, chemical synthesis of ncAA followed by its exogenous supplementation to the culture medium remains the most common strategy used for the production of ncAA-containing proteins. However, this is both costly and time-consuming, and in some cases, the ncAAs may be cell-impermeant, rendering them unavailable for translational incorporation. A much more cost-effective and straightforward approach is to biosynthesize the ncAA substrate in situ and genetically incorporated it into target proteins using a workhorse such as *E. coli*. For example, Thomas Carell et al.^39^ had demonstrated the use of wild type PylC and PylD enzymes to biosynthesize 3*S*-ethynylpyrrolysine (ePyl), a “clickable” pyrrolysine variant, from the chemically synthesized precursor 3*R*-ethynyl-d-ornithine, which is structurally similar to 3*R*-methyl-d-ornithine—the natural substrate of PylC. Taking advantage of the promiscuity of PylRS in substrate recognition and binding, ePyl was directly incorporated into chloramphenicol acetyltransferase and carbonic anhydrase 2 to produce the clickable enzymes—all accomplished within the *E. coli* cells. A different approach was taken by Budisa et al.^40^ who utilized an endogenous *E. coli* enzyme *O*-acetylserine sulfhydrylase to intracellularly synthesize *S*-allyl cysteine from inexpensive allyl mercaptan and an evolved *Mm*PylRS, SacRS, to genetically incorporated *S*-allyl cysteine into proteins. While these studies illustrate the use of in cellulo synthesized ncAAs for translational incorporation by the pyrrolysine translational machinery, our strategy is an advancement in this arena in that it can be potentially extended to multiple ncAAs by simply altering PylC recognition specificity.

In the field of drug development, macrocyclization is commonly employed to enhance the selectivity and potency of drug candidates. A case in point is lorlatinib, a FDA-approved synthetic macrocyclic kinase inhibitor for the treatment of NSCLC, that exemplifies the markedly improved pharmacological properties endowed by macrocyclization compared with its acyclic counterpart crizotinib.^41^ For cyclic protein therapeutics, which are among the largest molecules to be cyclized, the low cyclization efficiency of proteins with long amino acid chains remains a challenge to be overcome. In this report, we accomplished the macrocyclization of a 23-mer P16p in high yield and completion with our d-Cys-ε-Lys-based biosynthesis strategy. It is true that in general, chemical synthesis of linear peptides such as the P16p here is simpler since no DNA cloning and protein expression are required. However, producing cyclic peptides with the length of more than 20 amino acids in high purity is difficult to achieve. Chemical synthesis strategies, such as native chemical ligation^42^ (NCL) and copper(I)-catalyzed azide–alkyne cycloadditions (CuAAC) click reaction,^43,44^ are commonly used in peptide cyclization, but are mostly limited to about 20 amino acids. Therefore, the development of a facile cyclization method for long peptides is highly desirable. Besides, compared with standard NCL reaction, which relies on a fixed N-terminal cysteine, our facile cyclization was achieved by rational designed position of d-Cys-ε-Lys and the C-terminal thioester. According to our results, as they are placed in close proximity, the cyclization efficiency and completion were highly improved. Thus, our strategy might be particularly suitable for the macrocyclization of long peptides with preformed structure motifs, such as helix-turn-helix, β-strand, and β-hairpin motifs, or the intramolecular cyclization between protein subunits. Moreover, given the numerous structures of therapeutic targets that have been determined, one can imagine that this approach of generating cyclized fragments of relevant binding regions could be applied to multiple systems to produce active peptides whose reduced size helps to avoid immune responses.

## Conclusions

In conclusion, we have developed a highly efficient and robust platform based on the pyrrolysine technology for the production of cyclized proteins. Our system is distinct, in that the ncAA substrate is intracellularly synthesized from cheap and commercially available simple amino acids, which greatly reduces the cost of production of the target protein—an essential attribute in the development of therapeutic products. Via the optimization of the readthrough system and the implementation of a structure-inspired macrocyclization strategy, ncAA-bearing proteins can be produced in high yield and cyclized efficiently to near completion. With this optimized system, we successfully cyclized the P16 peptide subunit of a fusion protein under mild and reducing conditions within 3 h and showed that the cyclized P16p subunit exhibited enhanced binding affinity with CDK4 and a more potent inhibitory effect on pRb phosphorylation than its linear counterpart, resulting in meaningful G0/G1 arrest in MCF-7 cells. Given the important role of cyclic proteins/peptides in drug development, our incorporation system and cyclization strategy can potentially be applied to cyclize other potential therapeutic drug candidates, thereby providing a distinct and complementary approach in the preparation of cyclic peptide-containing proteins.

## Supporting Information Available

Linear P16p MD simulations (MPG)
Cyclic P16p MD simulations (MPG)
Experimental methods, crystal structure of the P16/CDK6 complex, schematic diagram of key coding region for plasmid constructs used in PylS evolution, computational analysis of the location of the triple mutations in M. mazei PylRS^EVF^ relative to the binding sites for tRNA^pyl^ and pyrrolysine, computational modeling of d-Cys-ε-Lys binding with PylC^NPSV^, ESI-MS/MS spectrum of d-Cys-ε-Lys-containing peptide fragment identified from tryptic peptides of GFP-X-P16p, schematic representation of constructs for P16p cyclization, cyclization time course of d-Cys-ε-Lys-containing proteins, cyclic P16 peptide interacts with CDK4, cellular uptake analysis of linear and cyclized cycRGD-mCh-P16p proteins by confocal microscopy, cell cycle arrest analysis with different P16p treatments at different time points, promoter sequences, and primer sequences (PDF)

## Supplementary Material

ja3c01291_si_001.mpg

ja3c01291_si_002.mpg

## References

[ref1] Sato A. K.; Viswanathan M.; Kent R. B.; Wood C. R. Therapeutic peptides: technological advances driving peptides into development. Curr. Opin. Biotechnol. 2006, 17, 638–642. 10.1016/j.copbio.2006.10.002. 17049837

[ref2] Colgrave M. L.; Craik D. J. Thermal, chemical, and enzymatic stability of the cyclotide kalata B1: the importance of the cyclic cystine knot. Biochemistry 2004, 43, 5965–5975. 10.1021/bi049711q. 15147180

[ref3] Ji Y.; Majumder S.; Millard M.; Borra R.; Bi T.; Elnagar A. Y.; Neamati N.; Shekhtman A.; Camarero J. A. In vivo activation of the p53 tumor suppressor pathway by an engineered cyclotide. J. Am. Chem. Soc. 2013, 135, 11623–11633. 10.1021/ja405108p. 23848581 PMC3767463

[ref4] Ngo K. H.; Yang R.; Das P.; Nguyen G. K.; Lim K. W.; Tam J. P.; Wu B.; Phan A. T. Cyclization of a G4-specific peptide enhances its stability and G-quadruplex binding affinity. Chem. Commun. 2020, 56, 1082–1084. 10.1039/c9cc06748e. 31894763

[ref5] Wilbs J.; Kong X.-D.; Middendorp S. J.; Prince R.; Cooke A.; Demarest C. T.; Abdelhafez M. M.; Roberts K.; Umei N.; Gonschorek P.; et al. Cyclic peptide FXII inhibitor provides safe anticoagulation in a thrombosis model and in artificial lungs. Nat. Commun. 2020, 11, 3890–3913. 10.1038/s41467-020-17648-w. 32753636 PMC7403315

[ref6] Clardy J.; Walsh C. Lessons from natural molecules. Nature 2004, 432, 829–837. 10.1038/nature03194. 15602548

[ref7] Driggers E. M.; Hale S. P.; Lee J.; Terrett N. K. The exploration of macrocycles for drug discovery—an underexploited structural class. Nat. Rev. Drug Discovery 2008, 7, 608–624. 10.1038/nrd2590. 18591981

[ref8] Deyle K.; Kong X. D.; Heinis C. Phage selection of cyclic peptides for application in research and drug development. Acc. Chem. Res. 2017, 50, 1866–1874. 10.1021/acs.accounts.7b00184. 28719188

[ref9] Tavassoli A.; Benkovic S. J. Split-intein mediated circular ligation used in the synthesis of cyclic peptide libraries in E. coli. Nat. Protoc. 2007, 2, 1126–1133. 10.1038/nprot.2007.152. 17546003

[ref10] Lee M. M.; Fekner T.; Lu J.; Heater B. S.; Behrman E. J.; Zhang L.; Hsu P. H.; Chan M. K. Pyrrolysine-inspired protein cyclization. Chembiochem 2014, 15, 1769–1772. 10.1002/cbic.201402129. 25044760

[ref11] Bouclier C.; Simon M.; Laconde G.; Pellerano M.; Diot S.; Lantuejoul S.; Busser B.; Vanwonterghem L.; Vollaire J.; Josserand V.; et al. Stapled peptide targeting the CDK4/cyclin D interface combined with Abemaciclib inhibits KRAS mutant lung cancer growth. Theranostics 2020, 10, 2008–2028. 10.7150/thno.40971. 32104498 PMC7019173

[ref12] Abdelkader E. H.; Qianzhu H.; George J.; Frkic R. L.; Jackson C. J.; Nitsche C.; Otting G.; Huber T. Genetic encoding of cyanopyridylalanine for in-cell protein macrocyclization by the nitrile-aminothiol click reaction. Angew. Chem., Int. Ed. 2022, 61, e202114154 10.1002/anie.202114154. PMC9304162 35102680

[ref13] Dong H.; Li J.; Liu H.; Lu S.; Wu J.; Zhang Y.; Yin Y.; Zhao Y.; Wu C. Design and ribosomal incorporation of noncanonical disulfide-directing motifs for the development of multicyclic peptide libraries. J. Am. Chem. Soc. 2022, 144, 5116–5125. 10.1021/jacs.2c00216. 35289603

[ref14] Dawson P. E.; Muir T. W.; Clark-Lewis I.; Kent S. B. Synthesis of proteins by native chemical ligation. Science 1994, 266, 776–779. 10.1126/science.7973629. 7973629

[ref15] Li X.; Fekner T.; Ottesen J. J.; Chan M. K. A pyrrolysine analogue for site-specific protein ubiquitination. Angew. Chem. 2009, 121, 9348–9351. 10.1002/ange.200904472. 19882608

[ref16] Serrano M.; Hannon G. J.; Beach D. A new regulatory motif in cell-cycle control causing specific inhibition of cyclin D/CDK4. Nature 1993, 366, 704–707. 10.1038/366704a0. 8259215

[ref17] Finn R. S.; Martin M.; Rugo H. S.; Jones S.; Im S. A.; Gelmon K.; Harbeck N.; Lipatov O. N.; Walshe J. M.; Moulder S.; et al. Palbociclib and letrozole in advanced breast cancer. N. Engl. J. Med. 2016, 375, 1925–1936. 10.1056/nejmoa1607303. 27959613

[ref18] Fåhraeus R.; Paramio J. M.; Ball K. L.; Laín S.; Lane D. P. Inhibition of pRb phosphorylation and cell-cycle progression by a 20-residue peptide from p16^CDKN2/INK4A^. Curr. Biol. 1996, 6, 84–91. 10.1016/s0960-9822(02)00425-6. 8805225

[ref19] Russo A. A.; Tong L.; Lee J.-O.; Jeffrey P. D.; Pavletich N. P. Structural basis for inhibition of the cyclin-dependent kinase Cdk6 by the tumour suppressor p16 ^INK4a^. Nature 1998, 395, 237–243. 10.1038/26155. 9751050

[ref20] Fujimoto K.; Hosotani R.; Miyamoto Y.; Doi R.; Koshiba T.; Otaka A.; Fujii N.; Beauchamp R. D.; Imamura M. Inhibition of pRb phosphorylation and cell cycle progression by an antennapedia-p16^INK4A^ fusion peptide in pancreatic cancer cells. Cancer Lett. 2000, 159, 151–158. 10.1016/s0304-3835(00)00536-x. 10996726

[ref21] Hosotani R.; Miyamoto Y.; Fujimoto K.; Doi R.; Otaka A.; Fujii N.; Imamura M. Trojan p16 peptide suppresses pancreatic cancer growth and prolongs survival in mice. Clin. Cancer Res. 2002, 8, 1271–1276. 11948142

[ref22] Camarero J. A.; Pavel J.; Muir T. W. Chemical synthesis of a circular protein domain: evidence for folding-assisted cyclization. Angew. Chem., Int. Ed. 1998, 37, 347–349. 10.1002/(sici)1521-3773(19980216)37:3<347::aid-anie347>3.0.co;2-5. 29711251

[ref23] Fan C.; Xiong H.; Reynolds N. M.; Soll D. Rationally evolving tRNA^Pyl^ for efficient incorporation of noncanonical amino acids. Nucleic Acids Res. 2015, 43, e156 10.1093/nar/gkv800. 26250114 PMC4678846

[ref24] Serfling R.; Lorenz C.; Etzel M.; Schicht G.; Böttke T.; Mörl M.; Coin I. Designer tRNAs for efficient incorporation of non-canonical amino acids by the pyrrolysine system in mammalian cells. Nucleic Acids Res. 2018, 46, 1–10. 10.1093/nar/gkx1156. 29177436 PMC5758916

[ref25] Kavran J. M.; Gundllapalli S.; O’Donoghue P.; Englert M.; Söll D.; Steitz T. A. Structure of pyrrolysyl-tRNA synthetase, an archaeal enzyme for genetic code innovation. Proc. Natl. Acad. Sci. 2007, 104, 11268–11273. 10.1073/pnas.0704769104. 17592110 PMC2040888

[ref26] Hohl A.; Karan R.; Akal A.; Renn D.; Liu X.; Ghorpade S.; Groll M.; Rueping M.; Eppinger J. Engineering a polyspecific pyrrolysyl-tRNA synthetase by a high throughput FACS screen. Sci. Rep. 2019, 9, 11971–11979. 10.1038/s41598-019-48357-0. 31427620 PMC6700097

[ref27] Nguyen D. P.; Elliott T.; Holt M.; Muir T. W.; Chin J. W. Genetically encoded 1, 2-aminothiols facilitate rapid and site-specific protein labeling via a bio-orthogonal cyanobenzothiazole condensation. J. Am. Chem. Soc. 2011, 133, 11418–11421. 10.1021/ja203111c. 21736333

[ref28] Pettersen E. F.; Goddard T. D.; Huang C. C.; Couch G. S.; Greenblatt D. M.; Meng E. C.; Ferrin T. E. UCSF Chimera—a visualization system for exploratory research and analysis. J. Comput. Chem. 2004, 25, 1605–1612. 10.1002/jcc.20084. 15264254

[ref29] Wannier T. M.; Kunjapur A. M.; Rice D. P.; McDonald M. J.; Desai M. M.; Church G. M. Adaptive evolution of genomically recoded Escherichia coli. Proc. Natl. Acad. Sci. 2018, 115, 3090–3095. 10.1073/pnas.1715530115. 29440500 PMC5866557

[ref30] Gaston M. A.; Zhang L.; Green-Church K. B.; Krzycki J. A. The complete biosynthesis of the genetically encoded amino acid pyrrolysine from lysine. Nature 2011, 471, 647–650. 10.1038/nature09918. 21455182 PMC3070376

[ref31] Quitterer F.; List A.; Beck P.; Bacher A.; Groll M. Biosynthesis of the 22nd genetically encoded amino acid pyrrolysine: structure and reaction mechanism of PylC at 1.5 Å resolution. J. Mol. Biol. 2012, 424, 270–282. 10.1016/j.jmb.2012.09.007. 22985965

[ref32] Brünger A. T.; Adams P. D.; Clore G. M.; DeLano W. L.; Gros P.; Grosse-Kunstleve R. W.; Jiang J.-S.; Kuszewski J.; Nilges M.; Pannu N. S.; et al. Crystallography & NMR system: A new software suite for macromolecular structure determination. Acta Crystallogr., Sect. D 1998, 54, 905–921. 10.1107/s0907444998003254. 9757107

[ref33] Brunger A. T. Version 1.2 of the crystallography and NMR system. Nat. Protoc. 2007, 2, 2728–2733. 10.1038/nprot.2007.406. 18007608

[ref34] Wadhwani P.; Afonin S.; Ieronimo M.; Buerck J.; Ulrich A. S. Optimized protocol for synthesis of cyclic gramicidin S: starting amino acid is key to high yield. J. Org. Chem. 2006, 71, 55–61. 10.1021/jo051519m. 16388617

[ref35] Yu Z.; Yu X. C.; Chu Y.-H. MALDI-MS determination of cyclic peptidomimetic sequences on single beads directed toward the generation of libraries. Tetrahedron Lett. 1998, 39, 1–4. 10.1016/s0040-4039(97)10485-3.

[ref36] Perlman Z. E.; Bock J. E.; Peterson J. R.; Lokey R. S. Geometric diversity through permutation of backbone configuration in cyclic peptide libraries. Bioorg. Med. Chem. Lett. 2005, 15, 5329–5334. 10.1016/j.bmcl.2005.07.089. 16213707

[ref37] Sugahara K. N.; Teesalu T.; Karmali P. P.; Kotamraju V. R.; Agemy L.; Girard O. M.; Hanahan D.; Mattrey R. F.; Ruoslahti E. Tissue-penetrating delivery of compounds and nanoparticles into tumors. Cancer Cell 2009, 16, 510–520. 10.1016/j.ccr.2009.10.013. 19962669 PMC2791543

[ref38] Ettinger A.; Wittmann T. Fluorescence live cell imaging. Methods Cell Biol. 2014, 123, 77–94. 10.1016/B978-0-12-420138-5.00005-7. 24974023 PMC4198327

[ref39] Ehrlich M.; Gattner M. J.; Viverge B.; Bretzler J.; Eisen D.; Stadlmeier M.; Vrabel M.; Carell T. Orchestrating the biosynthesis of an unnatural pyrrolysine amino acid for its direct incorporation into proteins inside living cells. Chem.—Eur. J. 2015, 21, 7701–7704. 10.1002/chem.201500971. 25845346

[ref40] Exner M. P.; Kuenzl T.; To T. M. T.; Ouyang Z.; Schwagerus S.; Hoesl M. G.; Hackenberger C. P.; Lensen M. C.; Panke S.; Budisa N. Design of S-allylcysteine in situ production and incorporation based on a novel pyrrolysyl-tRNA synthetase variant. Chembiochem 2017, 18, 85–90. 10.1002/cbic.201600537. 27862817

[ref41] Bauer T. M.; Shaw A. T.; Johnson M. L.; Navarro A.; Gainor J. F.; Thurm H.; Pithavala Y. K.; Abbattista A.; Peltz G.; Felip E. Brain penetration of lorlatinib: cumulative incidences of CNS and non-CNS progression with lorlatinib in patients with previously treated ALK-positive non-small-cell lung cancer. Targeted Oncol. 2020, 15, 55–65. 10.1007/s11523-020-00702-4. PMC7028836 32060867

[ref42] Zhang L.; Tam J. P. Synthesis and application of unprotected cyclic peptides as building blocks for peptide dendrimers. J. Am. Chem. Soc. 1997, 119, 2363–2370. 10.1021/ja9621105.

[ref43] Le Chevalier Isaad A.; Papini A. M.; Chorev M.; Rovero P. Side chain-to-side chain cyclization by click reaction. J. Pept. Sci. 2009, 15, 451–454. 10.1002/psc.1141. 19455541

[ref44] Jagasia R.; Holub J. M.; Bollinger M.; Kirshenbaum K.; Finn M. Peptide cyclization and cyclodimerization by Cu^I^-mediated azide-alkyne cycloaddition. J. Org. Chem. 2009, 74, 2964–2974. 10.1021/jo802097m. 19309103 PMC2677176

