# Supplementary material for: Pyrrolysine-Inspired in Cellulo Synthesis of an Unnatural Amino Acid for Facile Macrocyclization of Proteins
Source: J Am Chem Soc. 2023 Apr 26;145(18):10249–58. doi: 10.1021/jacs.3c01291 (PMC10176472; doi:10.1021/jacs.3c01291)
Supplement: Supplementary file 3 — ja3c01291_si_003.pdf [file ja3c01291_si_003.pdf]

Supporting Information

Pyrrolysine-inspired in cellulo synthesis of an unnatural amino acid for facile macrocyclization of proteins

Jingxuan Tai[a‡], Lin Wang[a‡], Wai Shan Chan[a‡], Jiahui Cheng[a], Yuk Hei Chan[a], Marianne M. Lee[a*], Michael K. Chan[a*]

[a] *School of Life Sciences and Center of Novel Biomaterials, The Chinese University of Hong Kong, Hong Kong SAR, China.*

‡These authors contributed equally to this work.

*Corresponding authors

Email: mariannemmlee@cuhk.edu.hk (M.M.L.); michaelkchan88@cuhk.edu.hk (M.K.C.)

Experimental Methods

Plasmids construction for directed evolution of PylRS

The plasmid pPylST-KanR(TAG)-mCh(TAG) used for the directed evolution of PylRS was derived from pPylST-mCh(TAG), which is a pETDuet-based plasmid harboring *pyl*S gene encoding PylRS (flanked by NcoI and BamHI sites) and *pyl*T gene encoding $tRNA^{Pyl}$ (flanked by XbaI site) from *Methanosarcina mazei,* as well as an mCherry gene with lysine codon at the 55th position mutated to TAG cloned between NdeI and KpnI sites.[1, 2] To produce the pPylST-KanR(TAG)-mCh(TAG) plasmid, a kanamycin resistance gene encoding aminoglycoside kinase containing a K42O mutation (i.e KanR(TAG)) was cloned upstream of the mCh(TAG) gene together with an intergenic spacer region containing an additional ribosome binding site (Supplementary Fig. 2b). A modified plasmid containing $tRNA^{M15}$ was generated by substituting the bases as indicated in the study of Serfling et al.[3] in the wild-type *pyl*T gene by performing two rounds of site-directed mutagenesis. The primers used were PylTm15_Fwd and PylTm15_Rev (Supplementary Table 2) for the 1st round of mutagenesis and PylTstarSecondC_Fwd and PylTstarSecondC_Rev for the 2nd round of mutagenesis.

PylRS library construction

To generate the PylRS mutant library, random mutations were introduced into the gene encoding PylRS and its variants by error-prone PCR (epPCR) using either the GeneMorph II Random Mutagenesis Kit according to the manufacturer's protocol, or *Taq* DNA polymerase with error-prone conditions referencing a previously reported protocol to achieve a mutation frequency of ~ 2.5 nucleotide mutations per amplicon.[4] The primers used were PylS Fwd and R4 which flanked the *pylS* gene. A PylRS mutant containing G14E and S451F mutations was subsequently identified. Since C348 has been reported to be critical for substrate binding, site saturation mutagenesis (SSM) was performed to randomize the C348 residue of the identified PylRS mutant using primers F2-PylS-Cys348 (with a degenerate NNN codon at C348) and R4. In each round of directed evolution, the gel-purified epPCR or SSM products were subcloned into the pPylST-KanR(TAG)-mCh(TAG) via megaprimer PCR of whole plasmids (MEGAWHOP) with reference to Miyazaki's protocol.[5] The resultant PCR products were treated with DpnI, followed by transformation into *E. coli* BL21(DE3) cells for library screening.

PylRS library screening

E. coli BL21(DE3) cells were transformed with the mutant library via heat shock or electroporation and allowed to recover by incubating at 37°C with shaking for 1 h. The transformed cells were spread on selection plates containing LB agar supplemented with 50 µM IPTG, 100 µg/mL ampicillin, 2 mM D-Cys-ε-Lys and 50 to 100 µg/mL kanamycin, and then incubated at 37°C overnight.

Positive clones from the kanamycin selection were re-streaked onto LB agar containing ampicillin followed by incubation at 37°C overnight. Fresh clones were then inoculated into wells containing 150 µL of LB medium supplemented with 100 µg/mL ampicillin and grew at 37°C with shaking at 230 rpm for 5 to 6 h. These cultures were then used in the 2nd screening based on mCherry fluorescence. Towards this end, 5 µL of each culture was added to a well of a 24-well plate containing 495 µL of LB medium or M9 glucose minimal medium (6 g/L Na_2HPO_4, 3 g/L KH_2PO_4, 1 g/L NH_4Cl, 0.5 g/L NaCl, 3 mg/L $CaCl_2$, 0.4% glucose and 1 mM $MgSO_4$ in MilliQ water) with supplementation of 50 µM IPTG, ampicillin and 2 mM D-Cys-ε-Lys, followed by incubation at 30°C overnight. 150 µL of each of the induced cultures was transferred to a black 96-well plate with clear bottom for measurements in a microplate reader (Tecan Infinite M1000 PRO). The normalized fluorescence intensity [measured fluorescence (Ex: 587 ± 5 nm; Em: 610 ± 5 nm) divided by the optical density at 700 nm] of each sample was used for selection. Cells that exhibited mCherry fluorescence stronger than the parent PylRS's were selected and cultured in 5 mL LB supplemented with ampicillin overnight for plasmid DNA extraction.

To validate that the selected PylRS mutants were true positives (i.e. do not use any endogenous amino acid as substrate and exhibit higher catalytic efficiency than the parent), the mCherry fluorescence screening was repeated in triplicate using freshly transformed colonies grown in LB medium supplemented with or without D-Cys-ε-Lys. A no ncAA control was included as a negative control.

Construction of pPylST.tL plasmid

To optimize the yield of the ncAA-containing protein, we employed the genetically recoded *E. coli* strain C321.ΔA.M9adapted that has been engineered to enhance nonstandard amino acid incorporation[6] for protein overexpression. This *E. coli* strain, however, is incompatible with the aforementioned pETDuet-derived pPylST vectors for two reasons. Firstly, the strain is ampicillin-

resistant, so ampicillin cannot be used as the selection marker for the mutant library transformants, and secondly, it does not produce T7 RNA polymerase that recognizes the T7 promoter in pETDuet-based plasmids, and which is essential for transcription. Thus, in order to utilize the C321.ΔA.M9adapted cells, several modifications were made to the pETDuet-based pPylST-mCh(TAG) vector bearing the $tRNA^{M15}$ and evolved $PylRS^{EVF}$ obtained from the directed evolution studies. In brief, these modifications involved (1) replacing the T7*lac* promoter located upstream of the 1st multiple cloning site (MCS1) (harboring $tRNA^{M15}$ and $PylRS^{EVF}$ genes) with the P_{tac} promoter; (2) substituting the T7*lac* promoter located upstream of the 2nd multiple cloning site (MCS2) (harboring a gene carrying an in-frame TAG codon) with the PL_{lacO1}promoter; and (3) exchanging the ampicillin resistance gene (*amR*) with a streptomycin/spectinomycin resistance gene (*smR*). The primers used to amplify the gene fragments for the aforementioned modifications are provided in Supplementary Table 2, and the amplicons were assembled using Gibson Assembly to build the resultant pPylST.tL-mCh(TAG) plasmid (Supplementary Fig. 2c).

mCherry readthrough assay

For evaluating the efficiency of different readthrough systems, *E. coli* competent cells (Rosetta 2(DE3) or C321.ΔA.M9adapted) cells were transformed with the relevant pPylST-mCh(TAG) or pPylST.tL-mCh(TAG) plasmid. The transformed cells were then inoculated into 50 mL of M9 medium containing appropriate antibiotics (ampicillin and/or spectinomycin) in a 96-well microtiter plate. After incubation at 30°C with shaking for 5 to 6 h, 250 μL of each culture was added into a 48-well plate. The medium was supplemented with the appropriate antibiotic, 0.5 mM IPTG and different concentration of ᴅ-Cys-ε-Lys. The plate was then incubated at 25 °C overnight with shaking. The mCherry fluorescence intensities of the induced cultures were measured as described above. The assay was performed in triplicate.

Generation of PylC mutant library by site-saturation mutagenesis

Site-saturation mutagenesis was performed on the residues S177, E179, D233 and T256 of PylC fused to an N-terminal SUMO tag to facilitate purification using two sets of degenerate primers (primers: PylC-S177E179mut_Fwd, PylC-D233mut_Rev, PylC-D233mut_Fwd and PylC-T256mut_Rev). The SUMO-PylC full-length fragment was extended by overlap extension PCR using

another two sets of non-mutated primers (primer: rbs-NdeI-SUMO_Fwd, PylC-S177up_Rev, PylC-T256down_Fwd, and PylC-KpnI-pDuet_Rev). The extended SUMO-PylC mutant insert was cloned into pACYCDuet-1 vector whose promotor was replaced with P_{lpp} to enable constitutive expression of PylC (primer: Plpp-rbs_Fwd). The reaction product yielded a PylC mutant library that could be screened in PylST expressing cells.

Screening of PylC mutant library

For screening of the PylC mutant library, BL21 (DE3) Star (Thermo Fisher) cells were transformed with plasmid pPylST.tL-KanR(TAG)-mCh(TAG). A single colony of the transformed cells was selected for the preparation of pPylST.tL-KanR(TAG)-mCh(TAG)-containing competent cells used for the subsequent transformation with the PylC mutant library by electroporation. The mutant library-transformed cells were plated on LB agar plates containing spectinomycin, chloramphenicol, 50 µM IPTG, 5 mM ᴅ-cysteine as well as different concentrations of kanamycin (150 and 200 µg/mL). Each transformation yielded approximately 68,300 transformants.

Colonies grew on the positive selection plates were transferred into 96-well microtiter plates containing 150 µL LB medium supplemented with chloramphenicol, spectinomycin, 50 µM IPTG and 5 mM ᴅ-cysteine for quantitation of the readthrough efficiency based on mCherry fluorescence as described above. The top 50 mutants that produced mCherry fluorescence exceeding that produced from wild-type PylC were selected for plasmid extraction for subsequent DNA sequencing for the identification of the corresponding mutation(s).

Computational modeling

Modeling of PylRS C348V was based on the crystal structure of *Mm*PylRS C-terminal domain (CTD) bound to adenylated pyrrolysine (PDB: 2ZIM).[7] The C348 residue was mutated to valine and the ligand was modified to adenylated ᴅ-Cys-ε-Lys in PyMOL[8]. Then the minimization of protein structure and analysis of surface hydrophobicity were performed using UCSF CHIMERA.[9]

The *Mm*PylRS and $tRNA^{pyl}$ complex model was manually built in PyMOL from two deposited structures: *Mm*PylRS NTD-tRNAPyl complex (PDB: 5UD5)[10] and *Mm*PylRS CTD bound with adenylated pyrrolysine (PDB: 2ZIM), using the *D.hafniense* PylRS CTD-tRNAPyl complex structure (PDB: 2ZNI)[11] as reference for alignment.

To generate the model of *Mm*PylC bound to D-Cys-ε-Lys, model *Mm*PylC bound to D-ornithine-ε-Lys was first build in the SWISS-MODEL[12] server taking the crystal structure of the *M. barkeri* $PylC^{WT}$ bound to D-ornithine-ε-Lys (PDB ID: 4FFM)[13] as a template. Then the D-ornithine-ε-Lys was replaced with D-Cys-ε-Lys in Pymol and the mutations (S177N, E179P, D233S, T256V) were incorporated into PylC chain. The final structure was refined by simulated annealing/molecular dynamics program from the CNS package.[14, 15] Binding and surface hydrophobicity analyses were performed using UCSF CHIMERA.

Molecular dynamics simulation of P16p

MD simulation of P16p was conducted using GROMACS software[16] version 2021.4 with opls2001 force field and TIP3P water model. The topology file of D-Cys-ε-Lys was generated using LigParGen server[17-19] in GROMACS format. The initial peptide was solvated by a dodecahedral water box with approximately 4000 water molecules and neutralized by adding Cl^- ions. The solvated system was minimized by steepest descent method using a tolerance of 1000 KJ/mol·nm and step size of 0.01 nm. The system was gradually heated from 0 to 298 K over 100 ps at the pressure of 1 bar. The production runs were carried out for 300 ns with a step size of 2 fs. The temperature was kept at 298 K by modified Berendsen thermostat with a time constant of 1 ps. The pressure was kept at 1 bar by Parrinello-Rahman scheme with a time constant of 2 ps and an isothermal compressibility of 4.5×10^{-5} bar^{-1}. Particle mesh Ewald (PME) method was employed to calculate long-range electrostatic interactions and a cut-off distance of 1 nm was used to calculate the short-range electrostatic and van der Waals interactions. The LINCS algorithm was employed to constrain all covalent bonds involving hydrogen atoms. Independent 300 ns simulations of both peptides were run 3 repeats from the same initial structure. Another 10 rounds of 10-ns simulation were run in the same condition for structure comparison. RMSD and RMSF analyses were performed using algorithms in GROMACS with least squares fit calculated based on backbone atoms.

Plasmid construction of proteins targeted for D-Cys-ε-Lys incorporation

The optimized plasmid pPylST.tL was used for subcloning different protein constructs for the subsequent cyclization studies. In brief, gene fragments encoding the protein of interest harboring the UAG codon and intein-CBD-His_7 tag were inserted in between KpnI and NdeI sites by Gibson

Assembly (NEB). For the construct cycRGD-mCh-cycP16p, an N-terminal SUMO (Small ubiquitin-like modifier protein) tag was included upstream to enhance protein expression. The linear counterparts of the D-Cys-ε-Lys incorporated proteins under study were subcloned similarly except the UAG codon in the gene fragment for cyclized proteins was replaced with GCG codon that encodes alanine by mutagenesis using Pfu Turbo DNA polymerase (Agilent Technologies) following manufacturer's instruction and the primers O-to-Ala-mutant_Fwd and O-to-Ala-mutant_Rev.

Protein expression, purification and cyclization of D-Cys-ε-Lys-containing proteins

For the expression of protein using chemically synthesized D-Cys-ε-Lys, the pPylST.tL plasmids harboring the protein constructs for cyclization (Supplementary Fig. 6) were transformed into *E. coli* C321 strain. The resultant transformants were inoculated into LB media supplemented with spectinomycin and grew at 30°C/220 rpm until OD_{600} reached 0.6. The cells were centrifuged to get rid of the culture media and the resultant cell pellets were resuspended in one-seventh of the original culture volume of 2×YT media supplemented with spectinomycin, induced with 0.5 mM IPTG and 4 mM D-Cys-ε-Lys for 16 h at 25 °C/220 rpm.

For the expression of protein using *in cellulo*-synthesized D-Cys-ε-Lys, the pACYCDuet-SUMO-$PylC^{NPSV}$ plasmid was co-transformed with the aforementioned pPylST.tL plasmids into *E. coli* C321 cells and cultured in 200 mL LB medium supplied with spectinomycin, chloramphenicol and 5mM D-cysteine (pH adjusted to 8.0 with 5 M Tris) at 30°C. When OD_{600} reached 0.6, the cell culture was centrifuged and resuspended in 50 mL LB medium, induced with 0.5 mM IPTG for 16 h at 25 °C/220 rpm.

At the end of the induction period, cells were harvested by centrifugation and resuspended in lysis buffer (20 mM Tris-HCl pH 8.0, 500 mM NaCl, 1 mM PMSF, 1 mM benzamidine). Cells were lysed by sonication on ice for 20 min and purified by Ni^{2+} affinity chromatography, except for SUMO-cycRGD-mCh-X-P16p-intein-CBD-His_7, which was purified using chitin resin to facilitate on-column cleavage by SUMO protease to remove the N-terminus SUMO tag and subsequent on-column cyclization as described below. Prior to cyclization, the purity of the purified protein was verified by SDS-PAGE.

Cyclization of the purified protein was achieved by the addition of 100 mM sodium 2-sulfanylethanesulfonate (MESNA) to initiate intein cleavage and 2mM tris(2-carboxyethyl) phosphine

(TCEP, pH adjusted to 8.0) to maintain a reducing environment. The cyclization process was performed in room temperature for 3 h, after which, the reaction mixture was incubated with chitin resin (New England Biolabs) to remove the cleaved intein-CBD-His_7, and the cyclized protein was collected from the mobile phase. An additional cyclization step to cyclize the N-terminal RGD of the cycRGD-mCh-X-P16p construct was performed in which the protein was subjected to air oxidation at 4 °C with gently shaking for 24 h.

The expression of the linear counterpart of the cyclized proteins was performed in transformed *E. coli* R2 strain cultured in LB media supplemented with 100 µg/mL ampicillin at 37°C/220 rpm until OD_{600} reached 0.6, at which point, 0.5 mM IPTG was used to induce expression for 16 h at 25°C/220 rpm. The same purification procedure described above for the corresponding cyclized proteins was used to purify the linear counterparts. To remove the intein-CBD-His_7, 50 mM DTT was added to the reaction mixture after which the intein-CBD-His_7 was similarly removed by chitin purification as previously described.

Electrospray ionization mass spectrometry

The protein band corresponding to GFP-X-P16p was excised, cut into 1 mm^3 pieces and destained by repeated wash steps using 50% MeOH/10 mM NH_4HCO_3, then dehydrated with ACN followed by vacuum drying. For protein reduction, 25 mM DTT was added and incubated at 56 ℃ for 1 h, followed by washing steps to remove remaining DTT before dehydration again. Trypsin was added to the dehydrated gel pieces and incubated at 37 ℃ overnight for digestion. Digested peptide was extracted from gel by sonication and the extracted samples were then separated by HPLC and analyzed on an Orbitrap Fusion Lumos Tribrid Mass Spectrometer (Thermo Fisher Scientific). For mass spectrometric analysis of intact protein, purified GFP-cycP16p was incubated with 25mM DTT for 24 h, followed by desalting using Bio-Gel P-30 size exclusion resin and denaturation by 0.1% formic acid before subjected to HPLC-MS analysis on an Orbitrap Fusion Lumos Tribrid Mass Spectrometer (Thermo Fisher Scientific). The capillary voltage was set to 3500 V. Spectra were acquired at a resolution of 120000 between 500-2000 m/z. Mass spectra were analyzed and deconvoluted by BioPharma Finder (Thermo Fisher Scientific) using Xtract algorithm.

Analytical size exclusion chromatography

To evaluate whether the cyclized P16p subunit on GFP-cycP16p will bind with CDK4, size analysis was performed on a mixture of GST-CDK4 (0.32 mg/mL, Sino Biological) and GFP-cycP16p (0.13 mg/mL) in 25 µL SEC running buffer (20 mM Tris pH 8.0, 200 mM NaCl) using Superdex 200 increase 5/150 GL analytical size exclusion column (Sigma-Aldrich) at a flow rate at 0.35 mL/min. Individual GST-CDK4 and GFP-cycP16p proteins were analyzed using the same condition as reference.

Binding studies

MicroScale Thermophoresis (MST) analysis was performed to measure the biomolecular interactions between GST-CDK4 (Sino Biological) and linear or cyclic MBP-P16p. The targets MBP-cycP16p or MBP-P16p were dialyzed against labeling buffer (20 mM Tris, 200 mM NaCl, pH 8.0) prior to labeling with Alexa Fluor 647 NHS ester dye (Thermo Fisher Scientific) at a molar ratio 1:10 (protein: dye) at RT for 2 h. Free dyes were removed using the gravity flow column B provided in the Monolith protein labeling Kit (Nanotemper). 16 sets of 1:1 serial dilution of the binding ligand GST-CDK4 was prepared with MST optimized buffer (20 mM Tris, 200 mM NaCl, 0.05% Tween 20, pH 8.0) while the concentration of the labeled targets was kept constant at 70 nM. The highest concentrations of GST-CDK4 used were 1.92 µM and 3.84 µM for MBP-cycP16p and MBP-P16p, respectively. Samples were mixed by pipetting and loaded into Monolith standard capillaries (NanoTemper) and their binding affinities were measured using the Monolith NT.115 with Nano-RED excitation type and the MST power set to medium. The collected data were processed using NanoTemper Affinity analysis software for the determination of the dissociation constant (Kd).

MCF-7 cell lysate pull down

MCF-7 cells were seeded at 4.0×10^5 cells per well in 6-well plates and incubated in RPMI-1640 medium supplemented with 10% FBS and penicillin/streptomycin (P/S) for 20 h. Cells were washed twice with ice-cold PBS and lysed in NP-40 lysis buffer (50 mM Tris, 150 mM NaCl, 2 mM EDTA, 1% NP-40, 0.1% SDS, pH 7.5) supplemented with protease inhibitor cocktail (Roche), followed by incubation in low temperature with gentle shaking for 20 min. Cell lysate was clarified by centrifugation at 15000 rpm for 15 min at 4°C. The total protein concentration of the supernatant was measured using Pierce™ BCA protein assay kit (Thermo Fisher Scientific) and was diluted to l.5 mg/mL using PBS.

This bait protein solution containing CDK4/6 was then incubated with purified MBP-cycP16p immobilized on amylose resin at 4°C for 3 h with gentle mixing. The reaction mixture was washed 5 times with PBS, after which the resin was analyzed by western blotting using anti-CDK4 (1:500, Biolegend) following standard protocol. The same bait protein solution was incubated with amylose resin only as a negative control.

Cellular uptake assay

MCF-7 cells in RPMI-1640 medium supplemented with 10% FBS and P/S (Cytiva) were seeded at 2.0×10^5 cells/mL in 35mm glass bottom confocal dishes (MatTek) and incubated at 37°C /5% CO_2 overnight for attachment. Next day the cells were washed with PBS to get rid of non-adherent cells and the adhered cells were starved for 24 h in serum free RPMI-1640 medium to induce cell cycle synchronization. Cells were then treated with 15 μM cycRGD-mCh-P16p or cycRGD-mCh-cycP16p for 24 h in RPMI-1640 medium supplemented with 10% FBS and P/S. Prior to confocal imaging, the nuclei and plasma membrane of the cells were counterstained with Hoechst 33342 (Thermo Fisher) and wheatgerm agglutinin (Thermo Fisher) respectively. Images were captured using a TCS SP8 Confocal Microscope (Leica) with excitation wavelengths set at 488, 561 and 633 nm.

Cell cycle arrest assay

MCF-7 cells were seeded in 12-well plates (1.5×10^5 cell per well) and incubated in RPMI-1640 medium supplemented with 10% FBS and P/S at 37°C. After 24-h attachment, cells were starved for another 24 h by replacing the medium with serum free RMPI-1640 medium. At the end of starvation period, the medium was changed back to RMPI-1640 supplemented with 10% FBS. Cells were then treated with 15 μM R_9-P16p, 15 μM cycRGD-mCh-P16p or 15 μM cycRGD-mCh-cycP16p, PBS (negative control), and 10 nM actinomycin (positive control) for different lengths of time (24, 48, 72 h). At the end of treatment period, cells were washed twice with PBS, trypsinized and fixed by 70% ethanol on ice for 45 min. The fixed cells were washed with PBS and stained by PI staining solution (10 μg/mL PI in PBS supplemented with RNase) for 30 min at RT in dark, followed by flow cytometric analysis using FACSVerse Flow Cytometer (BD Biosciences). Flow data was analyzed using ModFit LT 5.0 (BD Biosciences).

Cell proliferation assay

MCF-7 in RPMI-1640 medium supplemented with 10% FBS and P/S were seeded at 1.6×10^4 cells/well in a 96-well plate to allow for overnight attachment. Cells were then washed with PBS and treated with 5 - 20 μM cycRGD-mCh-P16p, cycRGD-mCh-cycP16p, or R_9-P16p (Pepmic) for 24 h. The number of viable cells was determined using the CellTiter 96 cell proliferation assay kit (Promega) following manufacturer's instruction and scanned on a Tecan Spark 10M Microplate Reader with the absorbance wavelength set at 490 nm. Data were normalized to the value of control cells treated with PBS. Experiment was repeated 3 times with similar results.

Western blot analysis

MCF7 cells were seeded at 4.0×10^5 cells per well in 6-well plates in RPMI-1640 medium supplemented with 10% FBS and P/S for overnight attachment. The medium was then changed to serum-free RPM-1640 medium and the cells were starved for 24 h. At the end of starvation, serum-free medium was replaced with RPMI-1640 supplemented with 10% FBS and P/S. 15 μM of cycRGD-mCh-P16p or cycRGD-mCh-cycP16p was added to the cells and incubated at 37°C / 5% CO_2 for 24 h. At the end of the treatment, cells were washed with ice-cold PBS and lysed in NP-40 lysis buffer (50 mM Tris, 150 mM NaCl, 2 mM EDTA, 1% NP-40, 0.1% SDS, pH 7.5) supplemented with protease inhibitor cocktail (Roche). Cell debris were removed by centrifugation at 15000 rpm for 10 minutes at 4°C. Samples were resolved by 10% SDS-PAGE, transferred to nitrocellulose membranes and probed using pRb (Ser780) antibody (1:1000, Cell Signaling), pRb (Ser795) antibody (1:500, Cell Signaling) and β-actin antibody (1:2500, Sigma-Aldrich). Proteins were visualized using the ECL system (Amersham).

Statistical analysis

Data from replicate experiments are presented as mean ± standard error (SD) of the mean. Statistical significance is noted in the figure legend where appropriate. For comparison of data in different groups, ordinary one-way ANOVA with Tukey's multiple comparison test was performed using GraphPad Prism 7 software (GraphPad, San Diego, USA). A p-value less than 0.05 is considered statistically significant. *p<0.05, ***p<0.001, ****p<0.0001, ns, not significant.

Supplementary Figures

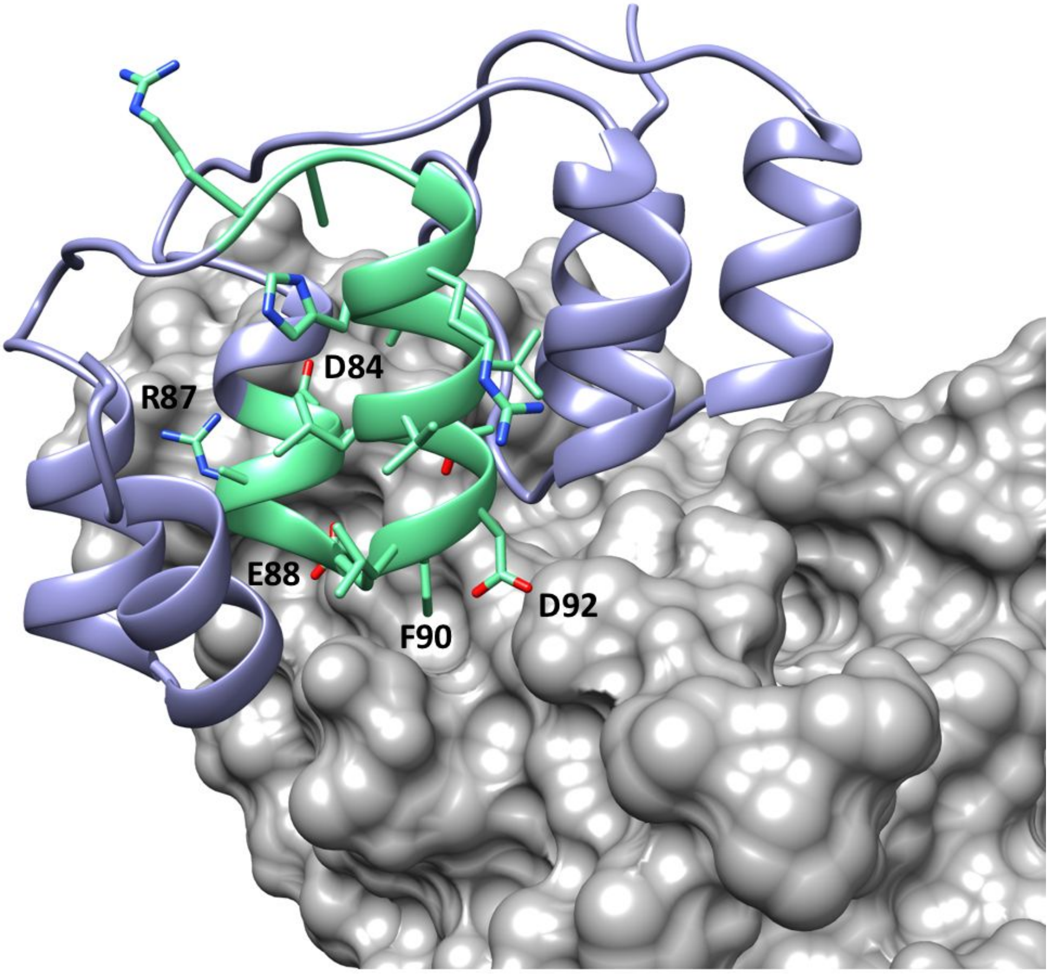

Supplementary Figure 1. Crystal structure of P16/CDK6 complex. Structure of P16 (blue) interacting with CDK6 (grey) derived from PDB: 1BI7.[20] The helix-turn-helix comprising P16p is highlighted in green with the residues interacting with CDK6 labeled.

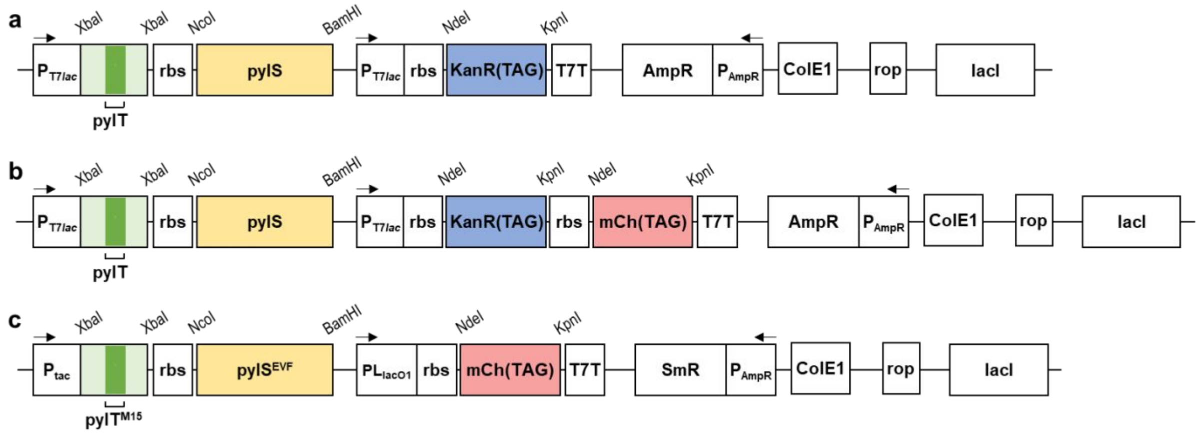

Supplementary Figure 2. Schematic diagram of key coding region for plasmid constructs used in PylS evolution. (a) pPylST-KanR(TAG) harboring single selection gene for first round PylRS screening. **(b)** pPylST-KanR(TAG)-mCh(TAG) harboring double selection gene for second and subsequent rounds PylRS screening. **(c)** pPylST.tL-mCh(TAG) harboring genes encoding $tRNA^{M15}$ and the evolved $PylRS^{EVF}$ used in the optimized readthrough system for efficient ᴅ-Cys-ε-Lys incorporation.

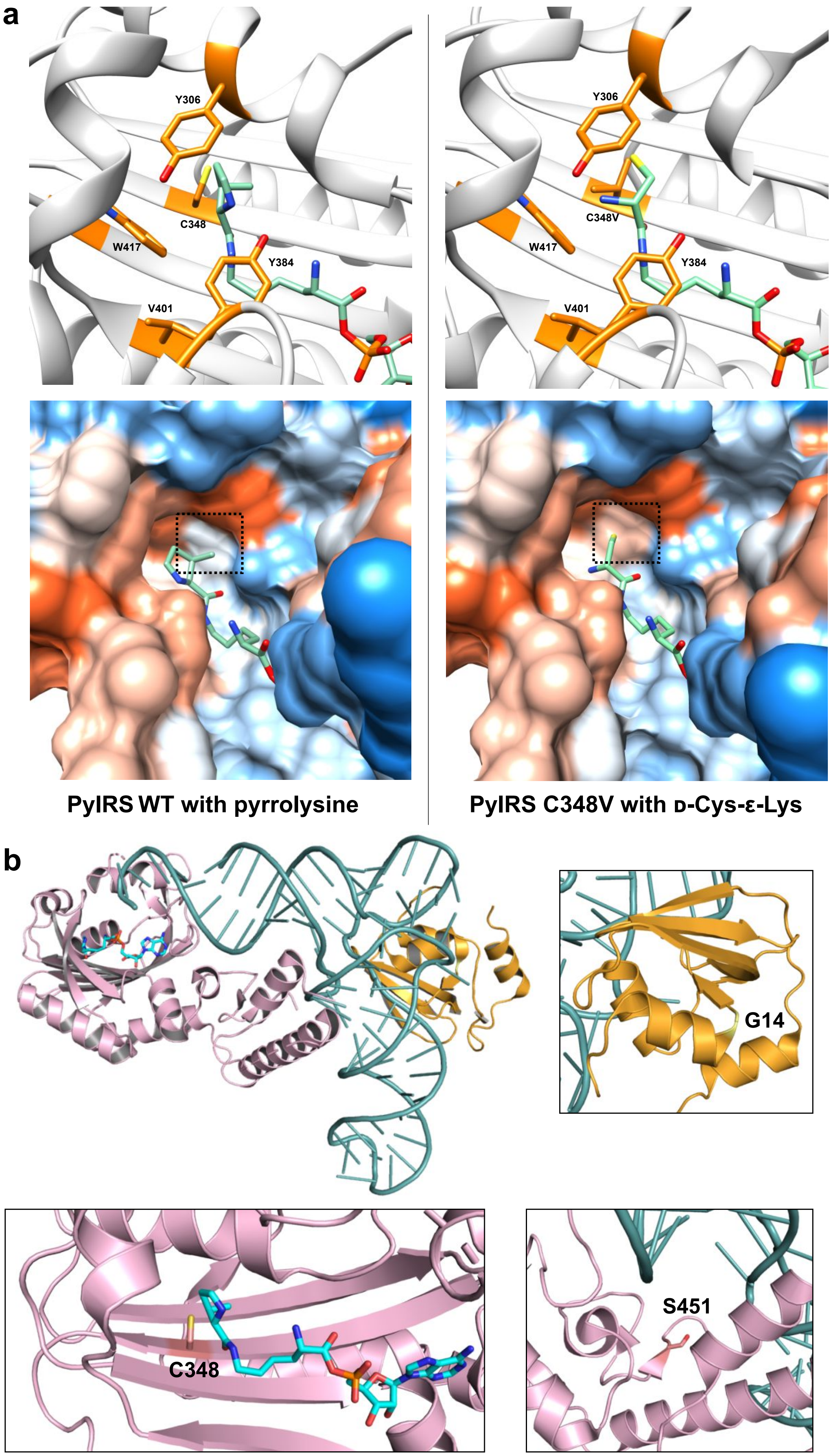
a
Y306
C348
W417
Y384
V401
Y306
C348V
W417
Y384
V401
PylRS WT with pyrrolysine
PylRS C348V with D-Cys-ε-Lys
b
G14
C348
S451

Supplementary Figure 3. Computational analysis of the location of the triple mutations in *M. mazei* PylRS^{EVF} relative to the binding sites for tRNA^{pyl} and pyrrolysine. (a) Comparison of the ligand binding site between crystal structure of *Mm*PylRS^{WT} (left) and computational model of *Mm*PylRS^{EVF} (right) bound to pyrrolysine and D-Cys-ε-Lys, respectively. Upper panels: Zoom-in view of pyrrolysine binding pocket in PylRS. The hydrophobic residues interacting with ligands are highlighted in orange. Pyrrolysine and D-Cys-ε-Lys are colored in pale green. Lower panels: Ligand binding pockets of PylRS and PylRS^{EVF} colored by surface hydrophobicity (blue for most hydrophilic, white for neutral, and orange-red for most hydrophobic). The positions of C348 or C348V are delineated with black boxes. **(b)** Modeled structure of wild type *Mm* PylRS bound to tRNA^{pyl} showing the locations of the G14E, C348V and S451F mutations in *Mm*PylRS^{EVF}.

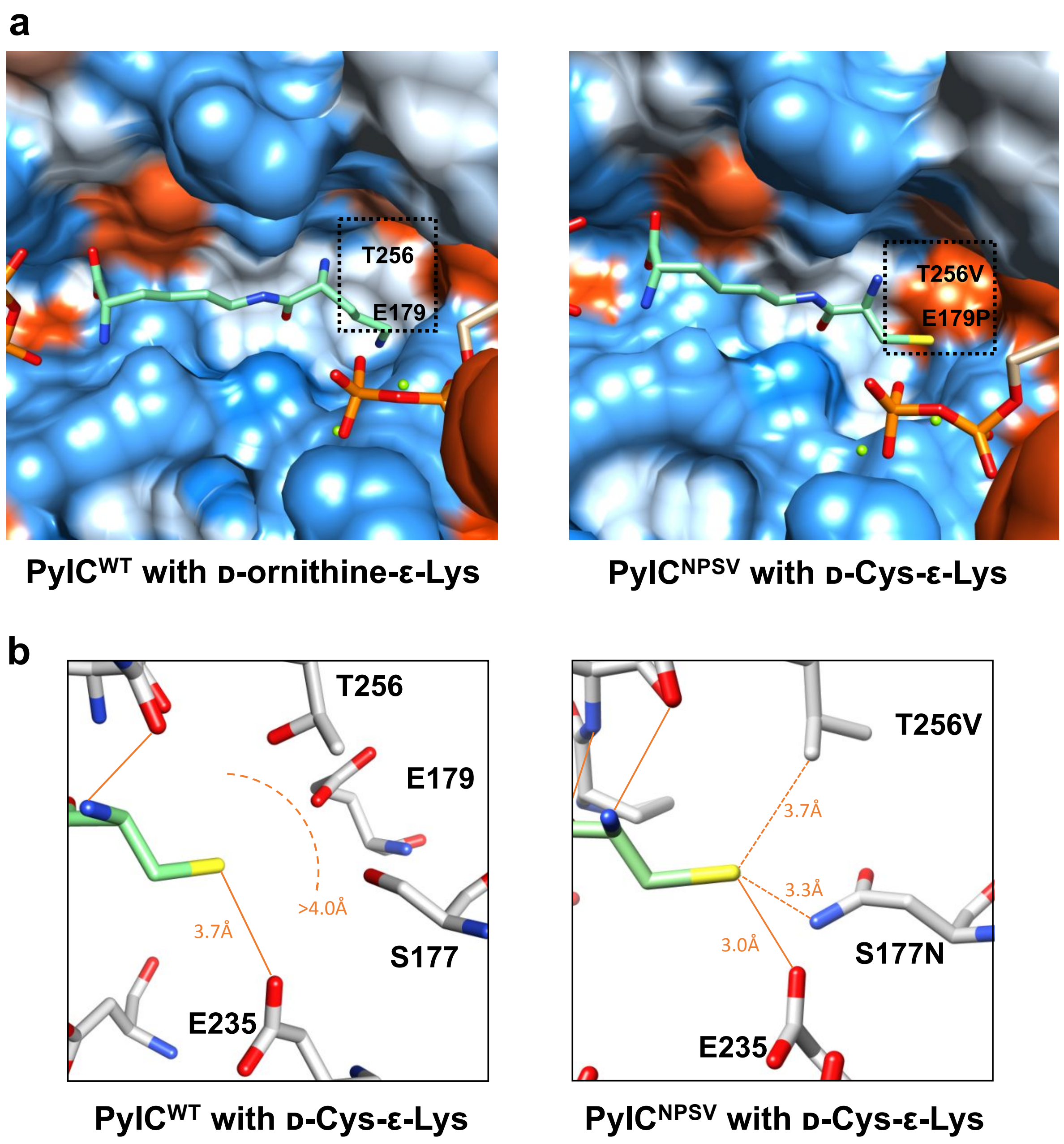

Supplementary Figure 4. Computational modeling of D-Cys-ε-Lys binding with PylC^{NPSV}. (a) Comparison of the surface hydrophobicity between crystal structure of PylC^{WT} (left) and computational model of PylC^{NPSV} (right) bound to D-ornithine-ε-Lys and D-Cys-ε-Lys, respectively. The surface of protein is colored according to its hydrophobicity (orange) or hydrophilicity (blue). D-ornithine-ε-Lys or D-Cys-ε-Lys was colored in pale green. Positions of residue 179 and 256 are delineated by black boxes. **(b)** Comparison of computational models of PylC^{WT} (left) and PylC^{NPSV} (right) bound to D-Cys-ε-Lys. H-bonds and Van der Waals interactions predicted by CHIMERA are shown as orange solid lines and dash lines, respectively.

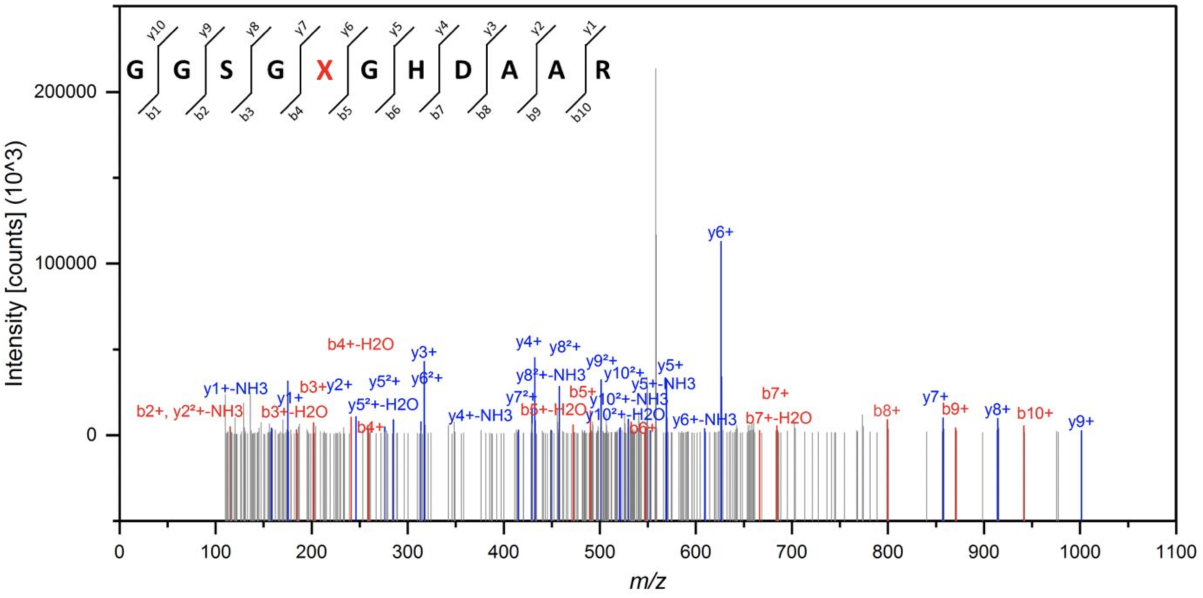

Supplementary Figure 5. ESI-MS/MS spectrum of D-Cys-ε-Lys-containing peptide fragment identified from tryptic peptides of GFP-X-P16p. GFP-X-P16p bearing the *in cellulo* biosynthesized D-Cys-ε-Lys was trypsin-digested and analyzed by mass spectrometry. Sequence of the peptide fragment containing D-Cys-ε-Lys is shown in the upper left corner with the collision-induced fragmentation pattern. X represents D-Cys-ε-Lys. The b and y ions are shown in red and blue, respectively.

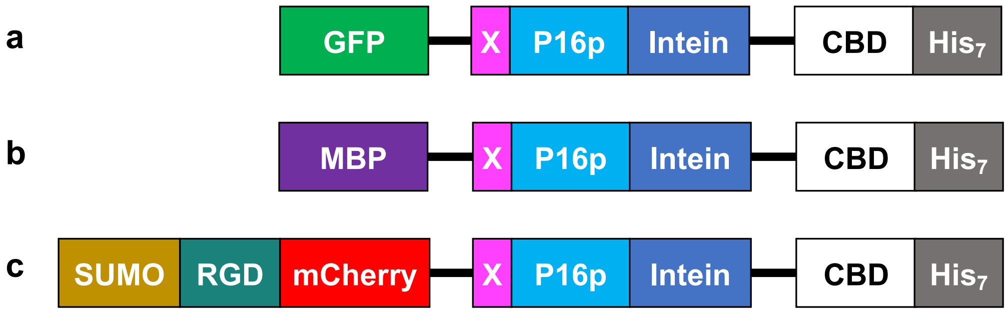

Supplementary Figure 6. Schematic representation of constructs for P16p cyclization. Protein constructs for production of **(a)** GFP-cycP16p, **(b)** MBP-cycP16p and **(c)** cycRGD-mCh-cycP16p are presented. X represents D-Cys-ε-Lys.

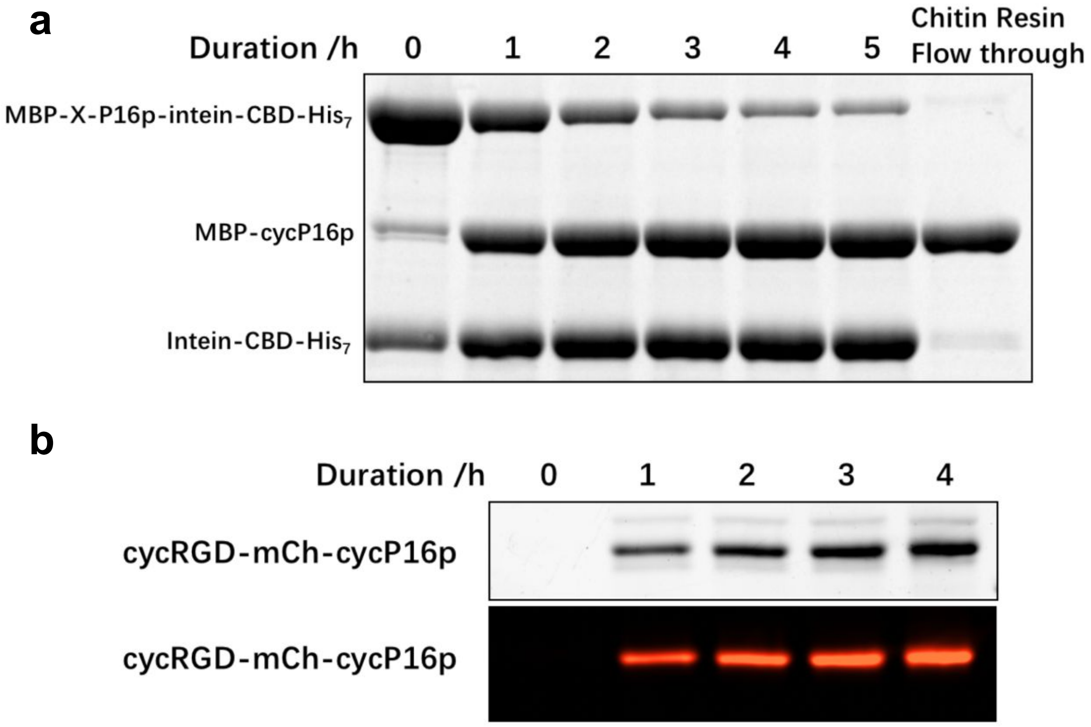

Supplementary Figure 7. Cyclization time course of D-Cys-ε-Lys-containing proteins. (a) SDS-PAGE of reaction sample taken at different time points during cyclization of MBP-X-P16p. The final product MBP-cycP16p was isolated from the reaction mixture by incubation with chitin resin to remove the CBD-tagged inteins. **(b)** SDS-PAGE of reaction samples taken at different time points during cyclization of cycRGD-mCh-cycP16p. Fluorescence imaging on a ChemiDoc Touch system (Biorad) was also used to detect mCherry fluorescence signal for validation (bottom).

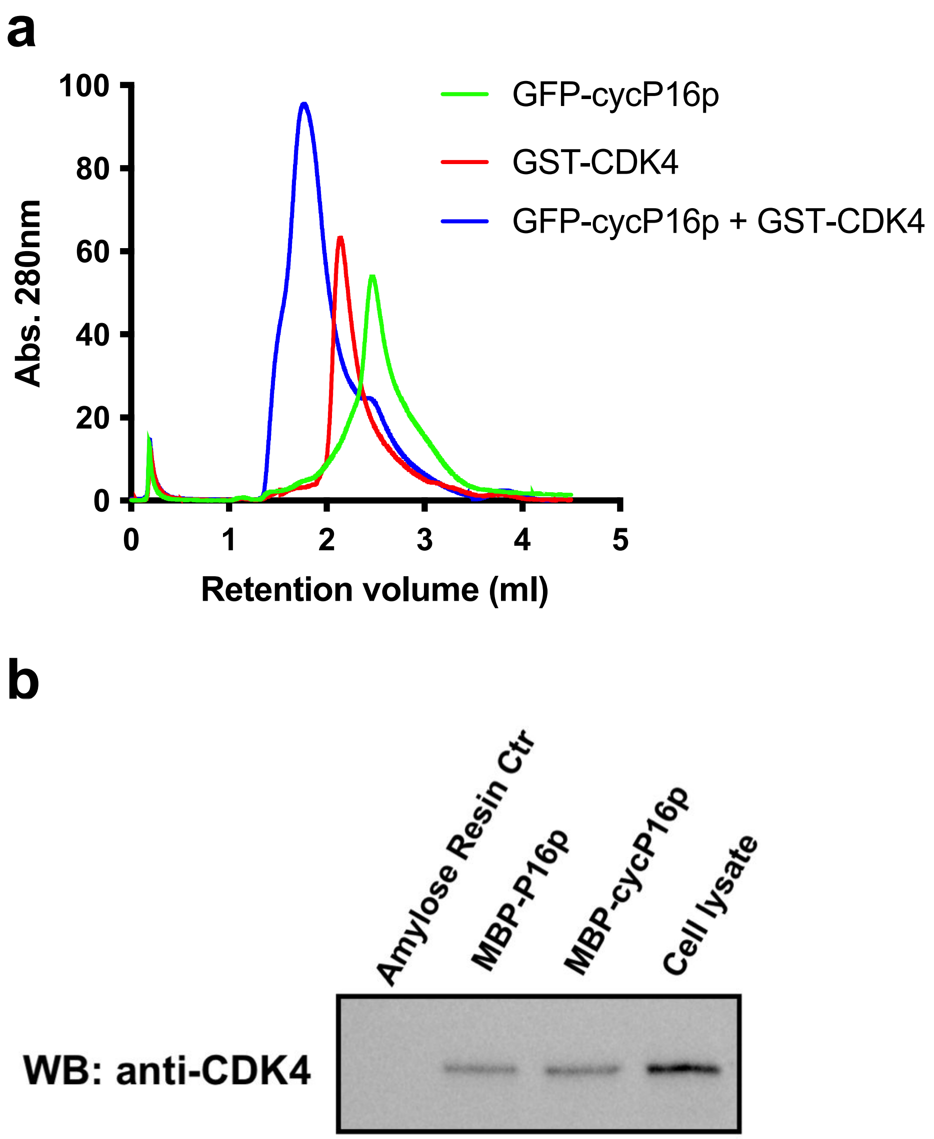

Supplementary Figure 8. Cyclic P16 peptide interacts with CDK4. (a) Characterization of GFP-cycP16p and GST-CDK4 complex by analytical size exclusion chromatography. The elution profile of the purified GFP-cycP16p (0.13 mg/mL) mixed with GST-CDK4 (0.32 mg/mL) in 25 μL SEC running buffer (20 mM Tris pH 8.0, 200 mM NaCl) (blue) was compared with the elution profiles of individual GST-CDK4 (red) and GFP-cycP16p (green) proteins analyzed using the same conditions. **(b)** Western-blot anti-CDK4 in MCF-7 cell lysate pull down assay. Amylose resin with pre-bound MBP-P16 peptides were incubated with MCF-7 cell lysate for pulling down CDK4 protein. Empty amylose resin as a negative control was also tested.

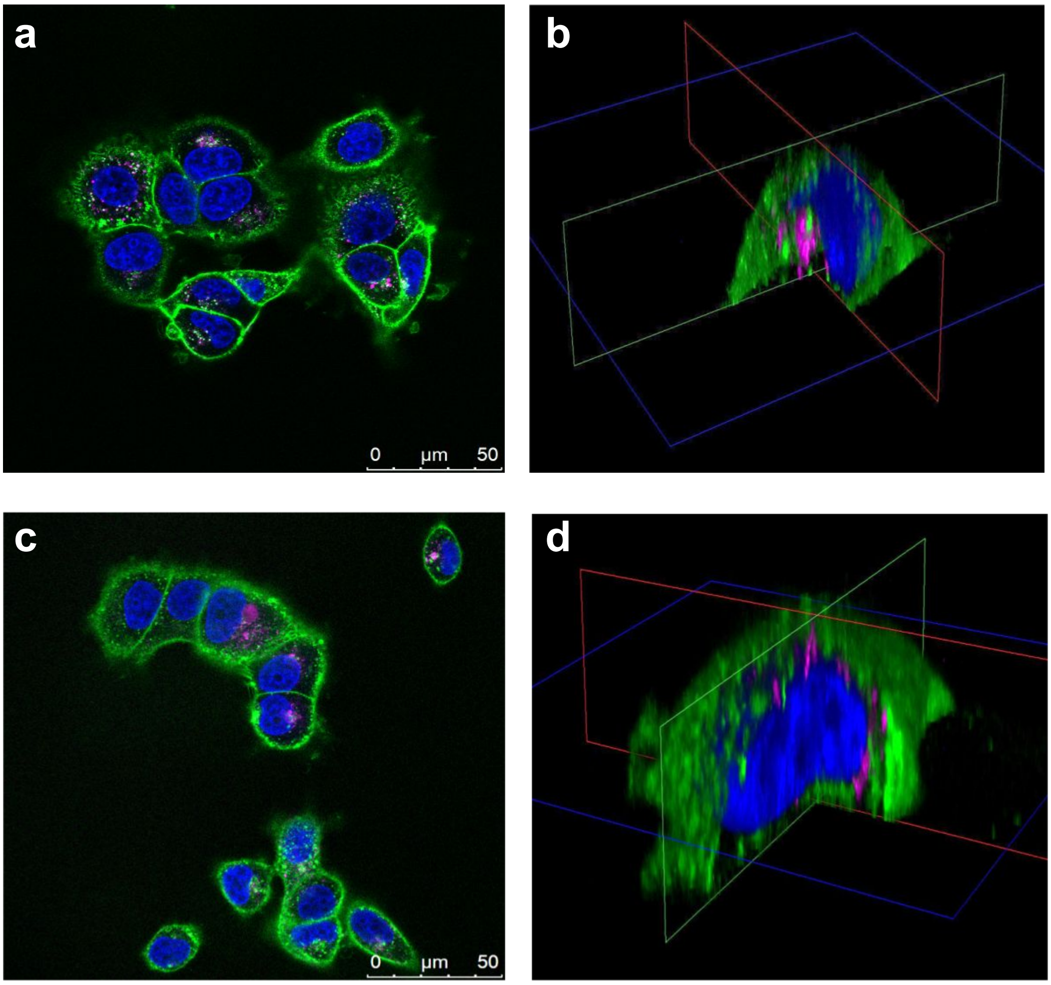

Supplementary Figure 9. Cellular uptake analysis of linear and cyclized cycRGD-mCh-P16p proteins by confocal microscopy. (a, b) Confocal images of MCF-7 cells treated with 15 µM cycRGD-mCh-P16p. **(c, d)** Confocal images of MCF-7 cells treated with 15 µM cycRGD-mCh-cycP16p. MCF-7 cell membranes were stained with wheat germ agglutinin (green), and the nuclei were counterstained with Hoechst 33342 (blue). Both linear and cyclic cycRGD-mCh-P16p can be detected within cells with fused mCherry tag (pink) (a, c). To clarify that the proteins were incorporated and not merely attached to the surface of the cells, sectional scanning was carried out using confocal laser scanning microscopy (b, d).

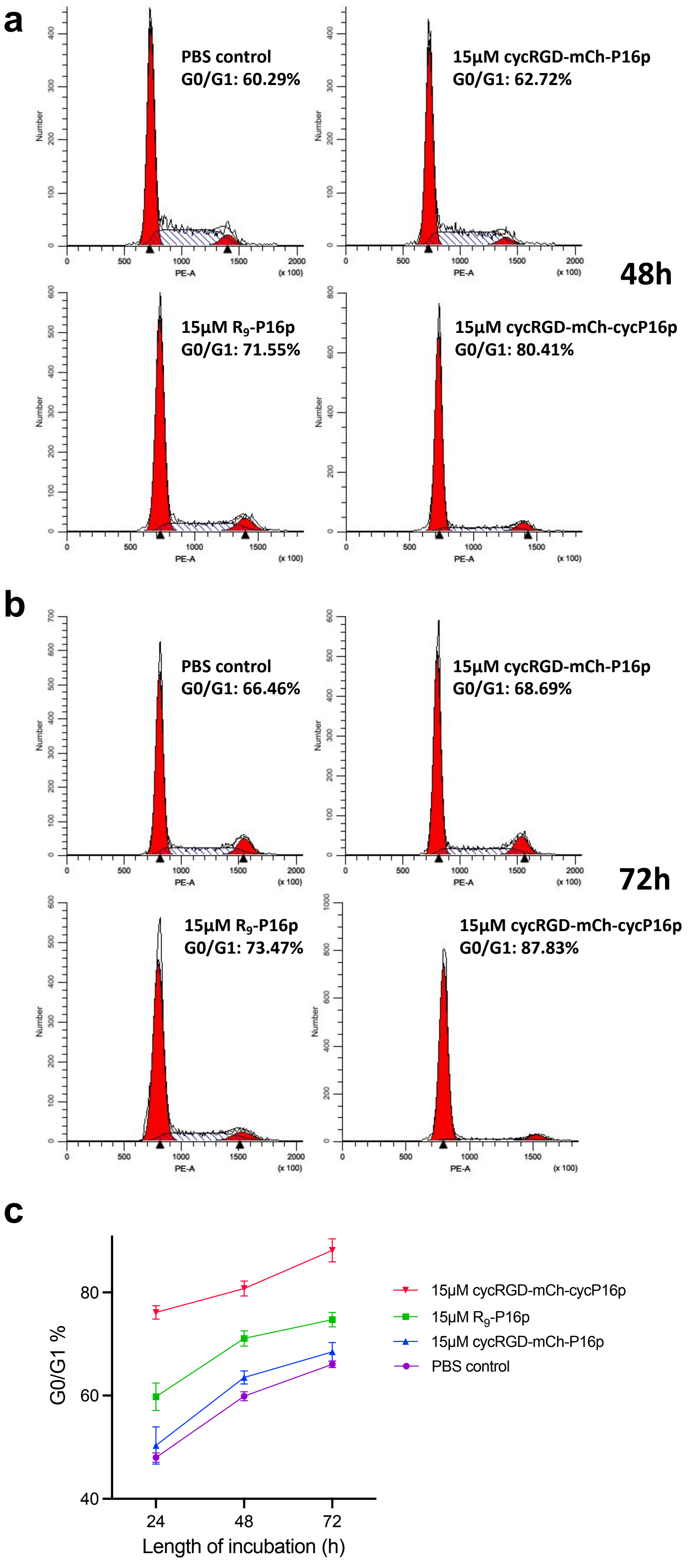
a
PBS control
G0/G1: 60.29%
15µM cycRGD-mCh-P16p
G0/G1: 62.72%
48h
15µM R9-P16p
G0/G1: 71.55%
15µM cycRGD-mCh-cycP16p
G0/G1: 80.41%
b
PBS control
G0/G1: 66.46%
15µM cycRGD-mCh-P16p
G0/G1: 68.69%
72h
15µM R9-P16p
G0/G1: 73.47%
15µM cycRGD-mCh-cycP16p
G0/G1: 87.83%
Number
PE-A
(x 100)
c
G0/G1 %
Length of incubation (h)
15µM cycRGD-mCh-cycP16p
15µM R9-P16p
15µM cycRGD-mCh-P16p
PBS control

Supplementary Figure 10. Cell cycle arrest analysis with different P16p treatments at different time points. (a) Representative results of MCF-7 cell cycle analysis by flow cytometry after 48-h treatment by different peptides. **(b)** Representative results of MCF-7 cell cycle analysis by flow cytometry after 72-h treatment by different peptides. (**c**) X-Y plot of the percentage of arrested MCF-7 cells in G0/G1 phase after exposure to different P16p treatments. Error bars present standard deviation (SD) of 3 replicate measurements.

Supplementary Tables

Supplementary Table 1. Promoter sequences:

P_{T7lac}	TAATACGACTCACTATAGGGGAATTGTGAGCGGATAACAATTCC
PL_{lacO1}	AATTGTGAGCGGATAACAATTGACATTGTGAGCGGATAACAAGATACTGAGCAC
P_{tac}	TTGACAATTAATCATCGGCTCGTATAATGTGTGGAATTGTGAGCGGATAACAA
P_{lpp}	CCCATCAAAAAAATATTCTCAACATAAAAAACTTTGTGTAATACTTGTAACGCT

Supplementary Table 2. Primer sequences:

Primer	Sequence	Application
PylS Fwd	CAGAGTCTATATCTAGAAATAATTTTGTTTAACTTTAAGAAGGAGATATACCATGG	EpPCR of PylS
R4	GCGCGCCGAGCTCGAATTCGGATCC	EpPCR of PylS
F2-PylS-Cys348	CATGCTGAACTTCNNNCAGATGGGATC	Site-saturation mutagenesis of PylS C348
PylTm15_Fwd	CAAGGAGGAAACCTGGTCAGGGAGACCGAACGGACTCTAAATC	Mutagenesis of $tRNA^{M15}$
PylTm15_Rev	GATTTAGAGTCCGTTCGGTCTCCCTGACCAGGTTTCCTCCTTG	Mutagenesis of $tRNA^{M15}$
PylTStarSecondC_Fwd	CCGTTCAGCCGGGTTCGATTCCCGGGGTTTCCGCCACGTTTC	Mutagenesis of $tRNA^{M15}$
PylTStarSecondC_Rev	GAAACGTGGCGGAAACCCCGGGAATCGAACCCGGCTGAACGG	Mutagenesis of $tRNA^{M15}$
PAmpRrev_Fwd	GGGTTATTGTCTCATGAGCGGATAC	Amplification of the pPylST.tL vector
DuetUP2-MCS2PLlacO1_Rev	CGCTCACAATGTCAATTGTTATCCGCTCACAATTATTTCG ATTATGCGGCCGTGTA	Amplification of the pPylST.tL vector
pDuet-MCS2Pdown_Fwd	CCATCTTAGTATATTAGTTAAGTA	Amplification of mCh(TAG)
pDuet-upAmpR_Rev	CTGTCAGACCAAGTTTACTCATATATACTTTAG	Amplification of mCh(TAG)
pDuet-SmRrev_Fwd	TAAACTTGGTCTGACAGTTATTTGCCGACTACCTTGGTG	Amplification of *smR* gene
PAmpRrev_Rev	GTATCCGCTCATGAGACAATAACCC	Amplification of *smR* gene
MCS1Ptac-PylT_Fwd	CGGCTCGTATAATGTGTGGAATTGTGAGCGGATAACAACC	Replacement of T7*lac* promoter with P_{tac}

rbs-NcoI-5PylS_Rev	CAGAGTGTTTAGTGGTTTTTATCCA TGGTATATCTCCTT CTTAAAGTTAAACA	Replacement of T7*lac* promoter with P_{tac}
pDuet-MCS1Ptac_Fwd	ATCTCGATCCCGCGAAATTTGACAAT TAATCATCGGCTCGTATAATGTGTGG A	Replacement of T7*lac* promoter with P_{tac}
PylC-S177E179mut_Fwd	GAATACGTTGAAGGGGAAGTGGTCN NKCTTNNKGTCATAGGGGATGGAAA TAATTTTGC	Generation of 4 PylC mutations
PylC-D233mut_Rev	GCCGGAAATCGCTTCCACMNNCATA ATTCCTTTTAAGGGCAGGTTTGC	Generation of 4 PylC mutations
PylC-D233mut_Fwd	GCAAACCTGCCCTTAAAAGGAATTA TGNNKGTGGAAGCGATTTCCGG	Generation of 4 PylC mutations
PylC-T256mut_Rev	GGAAGAATAATAGACCGCAGTCGGM NNCTGGCTCGGGAAACGG	Generation of 4 PylC mutations
rbs-NdeI-SUMO_Fwd	AAGTATAAGAAGGAGATATACATAT GTCTGACCAGGAGGCAAAAC	Extension of SUMO-PylC fragment
PylC-S177up_Rev	GACCACTTCCCCTTCAACGTATTC	Extension of SUMO-PylC fragment
PylC-T256down_Fwd	CCGACTGCGGTCTATTATTCTTCC	Extension of SUMO-PylC fragment
PylC-KpnI-pDuet_Rev	GGTTTCTTTACCAGACTCGAGGGTAC CTTATGCAGCAGCTCCGAAAC	Extension of SUMO-PylC fragment
Plpp-rbs_Fwd	CATAAAAAACTTTGTGTAATACTTGT AACGCTCCATCTTAGTATATTAGTTA AGTATAAGAAGGAGAT	Replacement of promoter to P_{lpp}
O-to-Ala-mutant_Fwd	ATACAAAGGAGGAAGTGGAGCGGGA CACGACGCTGC	Mutagenesis of TAG to GCG
O-to-Ala-mutant_Rev	GCAGCGTCGTGTCCCGCTCCACTTCC TCCTTTGTAT	Mutagenesis of TAG to GCG

References

1. Li, X.; Fekner, T.; Chan, M. K., N6-(2-(R)-propargylglycyl) lysine as a clickable pyrrolysine mimic. *Chem. Asian J.* **2010,** *5* (8), 1765-9.

2. Fekner, T.; Li, X.; Lee, M. M.; Chan, M. K., A pyrrolysine analogue for protein click chemistry. *Angew. Chem. Int. Ed.* **2009,** *48* (9), 1633-5.

3. Serfling, R.; Lorenz, C.; Etzel, M.; Schicht, G.; Böttke, T.; Mörl, M.; Coin, I., Designer tRNAs for efficient incorporation of non-canonical amino acids by the pyrrolysine system in mammalian cells. *Nucleic Acids Res.* **2018,** *46* (1), 1-10.

4. Wilson, D. S.; Keefe, A. D., Random mutagenesis by PCR. *Curr. Protoc. Mol. Biol.* **2000,** *51* (1), 8.3. 1-8.3. 9.

5. Miyazaki, K., MEGAWHOP cloning: a method of creating random mutagenesis libraries via megaprimer PCR of whole plasmids. *Meth. Enzymol.* **2011,** *498*, 399-406.

6. Wannier, T. M.; Kunjapur, A. M.; Rice, D. P.; McDonald, M. J.; Desai, M. M.; Church, G. M., Adaptive evolution of genomically recoded *Escherichia coli*. *Proc. Natl. Acad. Sci.* **2018,** *115* (12), 3090-3095.

7. Kavran, J. M.; Gundllapalli, S.; O'Donoghue, P.; Englert, M.; Söll, D.; Steitz, T. A., Structure of pyrrolysyl-tRNA synthetase, an archaeal enzyme for genetic code innovation. *Proc. Natl. Acad. Sci.* **2007,** *104* (27), 11268-11273.
8. DeLano, W. L. PyMOL molecular viewer: Updates and refinements, *Abstr. Pap. Am. Chem. Soc.*, **2009**; 238.
9. Pettersen, E. F.; Goddard, T. D.; Huang, C. C.; Couch, G. S.; Greenblatt, D. M.; Meng, E. C.; Ferrin, T. E., UCSF Chimera—a visualization system for exploratory research and analysis. *J. Comput. Chem.* **2004,** *25* (13), 1605-1612.
10. Suzuki, T.; Miller, C.; Guo, L.-T.; Ho, J. M.; Bryson, D. I.; Wang, Y.-S.; Liu, D. R.; Söll, D., Crystal structures reveal an elusive functional domain of pyrrolysyl-tRNA synthetase. *Nat. Chem. Biol.* **2017,** *13* (12), 1261-1266.
11. Nozawa, K.; O'Donoghue, P.; Gundllapalli, S.; Araiso, Y.; Ishitani, R.; Umehara, T.; Söll, D.; Nureki, O., Pyrrolysyl-tRNA synthetase–$tRNA^{Pyl}$ structure reveals the molecular basis of orthogonality. *Nature* **2009,** *457* (7233), 1163-1167.
12. Waterhouse, A.; Bertoni, M.; Bienert, S.; Studer, G.; Tauriello, G.; Gumienny, R.; Heer, F. T.; de Beer, T. A. P.; Rempfer, C.; Bordoli, L., SWISS-MODEL: homology modelling of protein structures and complexes. *Nucleic Acids Res.* **2018,** *46* (W1), W296-W303.
13. Quitterer, F.; List, A.; Beck, P.; Bacher, A.; Groll, M., Biosynthesis of the 22nd genetically encoded amino acid pyrrolysine: structure and reaction mechanism of PylC at 1.5 Å resolution. *J. Mol. Biol.* **2012,** *424* (5), 270-282.
14. Brünger, A. T.; Adams, P. D.; Clore, G. M.; DeLano, W. L.; Gros, P.; Grosse-Kunstleve, R. W.; Jiang, J.-S.; Kuszewski, J.; Nilges, M.; Pannu, N. S., Crystallography & NMR system: A new software suite for macromolecular structure determination. *Acta Crystallogr. D* **1998,** *54* (5), 905-921.
15. Brunger, A. T., Version 1.2 of the crystallography and NMR system. *Nat. Protoc.* **2007,** *2* (11), 2728-2733.
16. Abraham, M. J.; Murtola, T.; Schulz, R.; Páll, S.; Smith, J. C.; Hess, B.; Lindahl, E., GROMACS: High performance molecular simulations through multi-level parallelism from laptops to supercomputers. *SoftwareX* **2015,** *1*, 19-25.
17. Jorgensen, W. L.; Tirado-Rives, J., Potential energy functions for atomic-level simulations of water and organic and biomolecular systems. *Proc. Natl. Acad. Sci.* **2005,** *102* (19), 6665-6670.
18. Dodda, L. S.; Vilseck, J. Z.; Tirado-Rives, J.; Jorgensen, W. L., 1.14* CM1A-LBCC: localized bond-charge corrected CM1A charges for condensed-phase simulations. *J. Phys. Chem. B* **2017,** *121* (15), 3864-3870.
19. Dodda, L. S.; Cabeza de Vaca, I.; Tirado-Rives, J.; Jorgensen, W. L., LigParGen web server: an automatic OPLS-AA parameter generator for organic ligands. *Nucleic Acids Res.* **2017,** *45* (W1), W331-W336.
20. Russo, A. A.; Tong, L.; Lee, J.-O.; Jeffrey, P. D.; Pavletich, N. P., Structural basis for inhibition of the cyclin-dependent kinase Cdk6 by the tumour suppressor p16 INK4a. *Nature* **1998,** *395* (6699), 237-243.
